# Kinetics of the direct DME synthesis from CO_2_ rich syngas under variation of the CZA-to-γ-Al_2_O_3_ ratio of a mixed catalyst bed†

**DOI:** 10.1039/d1ra03452a

**Published:** 2021-07-13

**Authors:** Nirvana Delgado Otalvaro, Gerardo Sogne, Karla Herrera Delgado, Stefan Wild, Stephan Pitter, Jörg Sauer

**Affiliations:** Karlsruher Institute of Technology (KIT) Hermann-von-Helmholtz-Platz 1 D-76344 Eggenstein-Leopoldshafen Germany karla.herrera@kit.edu +49 721 608 28631

## Abstract

The one-step synthesis of dimethyl ether over mechanical mixtures of Cu/ZnO/Al_2_O_3_ (CZA) and γ-Al_2_O_3_ was studied in a wide range of process conditions. Experiments were performed at an industrially relevant pressure of 50 bar varying the carbon oxide ratio in the feed (CO_2_ in CO_*x*_ from 20 to 80%), temperature (503–533 K), space-time (240–400 kg_cat_ s m_gas_^−3^), and the CZA-to-γ-Al_2_O_3_ weight ratio (from 1 to 5). Factors favoring the DME production in the investigated range of conditions are an elevated temperature, a low CO_2_ content in the feed, and a CZA-to-γ-Al_2_O_3_ weight ratio of 2. A lumped kinetic model was parameterized to fit the experimental data, resulting in one of the predictive models with the broadest range of validity in the open literature for the CZA/γ-Al_2_O_3_ system.

## Abstract 2

Experimental and numerical kinetic investigations for the direct DME synthesis resulted in one of the predictive models with the broadest range of validity in the open literature for the CZA/γ-Al_2_O_3_ system.

## Graphical abstract

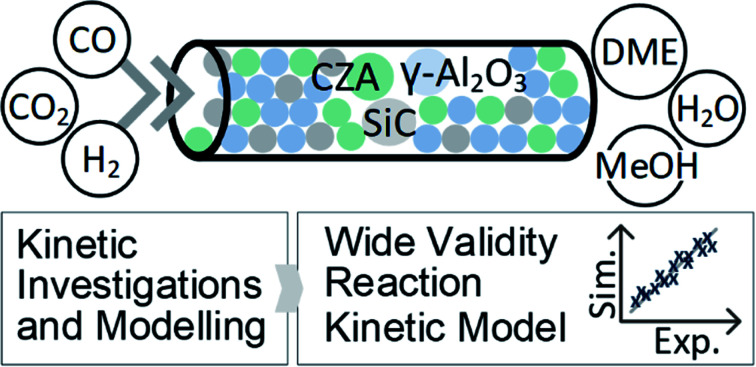

## 1. Introduction

Dimethyl ether (DME) has many uses in industries. Applications include its use as a coolant or a propellant, and as an important commodity for the production of lower olefins.^1^ Other potential applications include its use as a diesel substitute or fuel additive.^2,3^ Compared with fossil diesel fuels, the combustion of DME produces less NO_*x*_, CO, and particulate emissions, while still achieving a high performance with only minor modifications of the fuel storage and supply.^4,5^ DME is produced from synthesis gas, which originates from different sources such as coal, natural gas, and waste materials like biomass.^4–6^ Depending on the raw material and syngas production process, the composition of the syngas may change in a wide range, resulting in a variable feedstock for the DME synthesis.

The commercially established production route of DME involves two steps. The first step is methanol synthesis from syngas, followed by the methanol dehydration step in a second reactor. An alternative route is the direct or single-step synthesis, where DME is produced directly from syngas in a single reactor.^4^ Potential advantages of a single reactor are reduced complexity and investment costs. Also, the direct synthesis is thermodynamically advantageous compared to the conventional route.^7^ The *in situ* conversion of methanol by the dehydration reaction shifts the thermodynamic equillibrium of methanol synthesis towards the products. As a result, a higher conversion of the synthesis gas can be achieved under comparable conditions.^7^

Many dual catalyst systems have been proposed in the scientific literature for direct DME synthesis.^8–10^ These combine the properties of metallic catalysts for the methanol synthesis (typically copper-based),^11^ and a solid acid catalyst for the selective methanol dehydration to DME (such as γ-Al_2_O_3_, zeolites, and silica-modified alumina).^12^ In this contribution, we consider mechanical mixtures of the two commercial catalysts of each step *i.e.*, Cu/ZnO/Al_2_O_3_ (CZA) and γ-Al_2_O_3_.

Identifying and quantifying dependencies between process parameters and performance is essential for efficient, economically viable and safe process design and operation. Hence, numerous studies have been conducted investigating the influence of different variables on the performance of the direct DME synthesis from CO_2_ rich synthesis gas.

### CO_2_ content in the synthesis gas

Ateka *et al.*^13^ investigated the effect of CO_2_ content in the feed gas on the thermodynamics of the methanol and DME synthesis. Ng *et al.*^14^ studied the influence of CO_2_-to-CO_*x*_ ratios and catalyst bed compositions on the kinetics of the DME synthesis at 250 °C and 5 MPa. Peláez *et al.*^15^ described the effects of different feed gas compositions on the process performance at a pressure of 30 bar. These and other works^7,16–19^ have shown that increasing CO_2_ content in the feed decreases the process performance, and that water plays an important role, not only affecting the reaction kinetics, but also the catalyst structure by deactivation of the dehydration component γ-Al_2_O_3_.

### Catalyst bed composition and configuration

With regard to the composition of the catalyst bed, previous investigations^14,20–23^ have shown on the basis of simulated and experimental data that optimization can lead to significant enhancement of the process performance. For instance, in the studies of Peláez *et al.*^15^ and Peinado *et al.*^24^ the authors showed that for CO_2_ rich synthesis gas a significant increase in the performance is achieved by increasing the CZA-to-γ-Al_2_O_3_ ratio. In a previous study,^21^ applying a dynamic optimization scheme and experimental validation we showed that these effects hold true also for high pressure (50 bar) and different compositions of CO_2_ rich syngas, including a hydrogen-lean feed. Other studies^20,21,25^ on the loading and arrangement of physical catalyst mixtures have shown that homogeneously mixed catalyst beds achieve similarly good process performance compared to more complex configurations.

### Quantification and prediction of system behavior

Reliable models able to predict the process performance in different operating windows are necessary to enable the optimal reactor and process design, especially if DME synthesis is to be conducted at dynamic conditions or changing feed compositions. Therefore, several kinetic models have been proposed in the open literature to quantitatively describe and predict the effects of process variables on process performance. A widely used modelling approach is the combination of available models for the methanol synthesis,^26,27^ and its dehydration.^28^ Models derived for the direct DME synthesis under mechanistic assumptions include the works of Lu *et al.*,^29^ Aguayo *et al.*,^30^ Ereña *et al.*,^31^ and Peláez *et al.*^15^

Although so many studies have been carried out for the direct DME synthesis from CO_2_ rich synthesis gas, the detail reaction mechanism is still controversial.^32^ Therefore, reliable kinetic models valid in a wide range of conditions at industrially relevant process conditions are still necessary. In this work, we develop a reaction kinetic model applicable for an extended range of catalyst bed compositions, and process parameters (CO_2_ content in the synthesis gas, temperature and space time), extending the scope of available reaction kinetic models and providing a useful tool for model-based reactor and process design and optimization.

## 2. Experimental setup and procedures

In this chapter the equipment and methodology for the experimental kinetic investigations are described. First, the laboratory setup is described, then the materials used are listed, followed by a brief description of the experimental procedures and conditions at which the kinetic measurements were conducted.

### 2.1. Reactor and periphery

The reactor setup used in this work is presented in detail elsewhere.^21^ It consists of a laboratory tube reactor made of the stainless steel with an internal diameter of 12 mm, and a total length of 460 mm. The reactor is divided in four independent heating zones, each of which is surrounded by brass jaws equipped with heating cartridges (Horst GmbH) to set the temperature at the reactor outer wall. The gas supply is regulated *via* mass flow controller (Bronkhorst High-Tech B.V.) by using proportional integral derivative control. The system pressure is set by using a mechanical pressure regulator (Emerson Electric Co.). A gas chromatograph G1530A (Agilent Technologies) was used to analyse the composition of the feed gas and product gas.

### 2.2. Materials

Commercial catalysts, *i.e.*, Cu/ZnO/Al_2_O_3_ (CZA) and γ-Al_2_O_3_ (Alfa Aesar) were used as hydrogenation catalyst for the methanol synthesis and methanol dehydration to DME, respectively. Relevant properties of the used catalysts are provided in Table 1. The catalysts were ground and sieved to a particle size between 250 and 500 μm. To avoid hot spot formation, the catalytic bed was diluted with silicon carbide (SiC, Hausen Mineraliengroβhandel GmbH) of the same size distribution.

Selected properties of the commercial catalysts

**Table:** 

Properties of the CZA catalyst^10^	
Metal composition (Cu/Zn/Al)/wt%	64/29/6
Specific surface area (S_BET_)/m^2^ g^−1^	98
Pore volume/cm^3^ g^−1^	0.332
Maximum pore diameter/nm	11
Pore size range/nm	5–26

**Table:** 

Properties of the γ-Al_2_O_3_ catalyst^33^	
Specific surface area (S_BET_)/m^2^ g^−1^	213
NH_3_-TPD peak position in low and high temperature regions/K	512 and 624
Total acidity/mmol NH_3_ per g_cat_ (desorbed NH_3_ in NH_3_-TPD)	0.37
Acidity in low and high temperature regions/mmol NH_3_ per g_cat_	0.18 and 0.19

The feed gases, carbon monoxide (CO, 99.97%), nitrogen (N_2_, 99.9999%), hydrogen (H_2_, 99.9999%) and a mixture carbon dioxide/nitrogen (CO_2_/N_2_, 50 : 50 ± 1.0%) were purchased by Air Liquid Germany GmbH.

### 2.3. Kinetic measurements

Before performing the kinetic measurements, the CZA share of the catalytic bed was reduced at atmospheric pressure (5% H_2_ in N_2_, at temperatures between 393 and 513 K). Following the reduction procedure, the catalyst was conditioned until stable catalyst activity was achieved, in order to decouple the kinetic measurements from deactivation effects. The reduction and conditioning procedures are described in detail elsewhere^17^ and summarized in the ESI.† The kinetic measurements were performed at a pressure of 50 bar under variation of the CZA-to-γ-Al_2_O_3_ weight ratio (*μ*), temperature (*T*), space time (*τ*), and carbon oxide ratio (COR),1
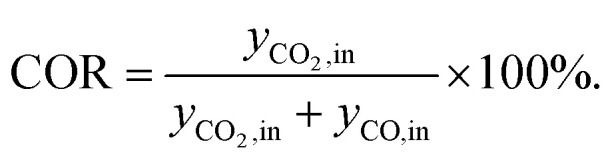


The experimental conditions as summarized in Table 2 were chosen in order to measure intrinsic kinetics *i.e.*, by minimizing heat and mass transport limitations. The total catalyst mass in all experiments was 2 g, while the mass of each catalyst was distributed in different ratios (*μ* = *m*_CZA_/*m*_γ-Al_2_O_3__). The mole fraction of H_2_ in the feed (*y*_H_2_,in_) was set to 46.5% to avoid a stoichiometric limitation in all cases. The mole fraction of carbon oxides in the feed, *i.e.*, *y*_CO_*x*_,in_ = *y*_CO_2_,in_ + *y*_CO,in_ was at 15%, and the fraction of N_2_ (*y*_N_2_,in_) was set accordingly to 38.5%. The concentrations used for the model parametrization were determined from the mean value of at least 4 chromatograms per operating point. Each set point was held for at least 3 hours enabling multiple readings, and confirmation of stability.

**Table:** Conditions for kinetic measurements

Variable	Values
Temperature (*T*), K	503, 513, 523, 533
Space-time^a^ (*τ*), kg_cat_ s m_gas_^−3^	240, 300, 400
Carbon oxide ratio (COR), %	20, 40, 60, 80
Catalyst ratio (*μ*), g_CZA_ g_γ-Al_2_O_3__^−1^	1, 2, 3, 5

a At standard conditions: *p* = 101 325 Pa, *T* = 293.15 K.

### 2.4. Estimation of model-specific parameters

The Matlab® (Version R2019a) built-in solver *ode45* was used to integrate the system of differential equations (Section 3.1) along the reactor axial coordinate. The model-specific parameters were fitted to experimental data using the nonlinear least-squares solver *lsqcurvefit* and the algorithm *trust-region-reflective*. The model-specific parameters were estimated such as to minimize the weighted sum of squared errors,2
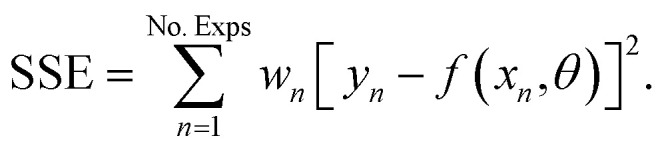
where *y*_*i*_ represent the response values (measured quantities), *f*(*x*_*n*_,*θ*) the predicted values with the nonlinear model function, and *x*_*n*_ and *θ* are respectively the predictor values of observation *n*, and the model-specific parameters.

The parameter estimation took place based on the measured mole fractions of the components in the product gas, excluding water and methanol since it was not possible to detect these species accurately over the wide range of conditions shown in Table 2. Reported values for water and methanol correspond to those calculated based on the component balances (C, H and O balance). Additionally, experimental data for which the component balances exhibited a relative error higher than 8% were excluded from the parameter estimation (*w*_*n*_ = 0). Due to the strong influence of initial parameter values, and in order to avoid local optimality, the fitting procedure was iteratively repeated until the relative difference between the parameters obtained in two consecutive iterations was lower than 5%. The Matlab built-in function *nlparci* was used to calculate the 95% confidence intervals of the parameter estimates using the residuals and the Jacobian matrix of the fitted model, which are both output arguments of *lsqcurvefit*. Additionally, correlation coefficients were computed using eqn (3),^34^3
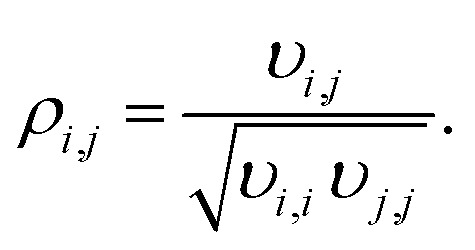
Here, *υ*_*i*,*j*_ represents the elements of the covariance matrix of the parameters of the fitted model. The covariance matrix *V*_*θ*_ is calculated with the variance of the experimental fluctuations *s*^2^ (assumed to be constant over all experiments) and the Jacobian matrix *J* by,

4 *V*_*θ*_ = *s*^2^(*J*^T^*J*)^−1^.

Correlation coefficients |*ρ*_*i*,*j*_| ≥ 0.95 are assessed to indicate a strong parameter correlation.^35^

## 3. Mathematical model

In this section, the mathematical model consisting of the reactor model (balance equations) and the reaction kinetic model (rate expressions) is presented.

### 3.1. Reactor model

The change of the mole fraction of the components along the reactor's axial coordinate can be described by the balance equation of an ideal plug flow reactor (eqn (5)). This simplified form of the general material balance of a fixed-bed reactor is admissible for the characteristics of the lab-scale reactor, and the conditions at which it was operated. Isothermal operation was achieved by diluting the catalyst bed with silicon carbide (SiC), and diluting the feed gas with inert N_2_. Temperature gradients did not exceed 2 K in any of the measurements. Hence, the assumption of isothermal operation applies and the energy balance can be omitted. All measurements took place under steady state conditions, which was verified experimentally. Furthermore, it was proven by the means of *a priori* criteria, that no significant influence of mass or heat transport processes took place, and that the assumption of plug flow applies. Finally, the pressure drop in the fixed bed was determined to be negligible by the means of correlations. Values to support the mentioned assumptions are reported in Table S1 in the ESI.† It can be concluded that the intrinsic reaction rates were measured in all experiments and that the reactor can be described by the balance equations of an ideal plug flow reactor. Furthermore, the volume contraction caused by reaction can be accounted for by eqn (6).5
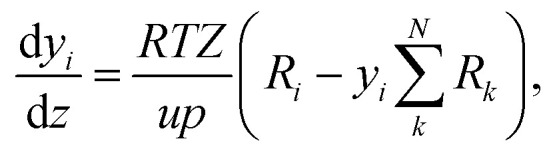
6
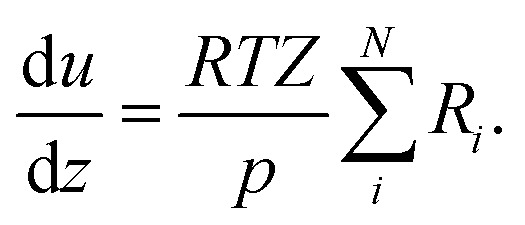
In eqn (5) and (6), *y*_*i*_ is the mole fraction of component *i*, *z* represents the position in the axial coordinate, *R* is the universal gas constant in J mol^−1^ K^−1^, *T* is the temperature in K, *p* is the pressure in Pa, *u* is the gas velocity in m s^−1^, *ϑ*_*i*,*j*_ is the stoichiometric coefficient of component *i* in reaction *j*, and *N* is the number of components in the system. *Z* is the compressibility factor of the mixture, which takes into account possible deviations from the ideal gas behavior at the high pressure (50 bar) considered in our investigations. The Peng–Robinson equation of state (PR-EoS)^36^ was chosen to calculate *Z*, since it has already been successfully applied to the system under consideration,^21,37^ and it provides accurate calculations for light gases, alcohols and hydrocarbons.^38^ In addition, van der Waals mixing rules^36^ were used to account for inter-molecule interactions. The molar rate of depletion or formation of component *i* due to chemical reaction (*R*_*i*_ in mol m^−3^ s^−1^) is defined by:7
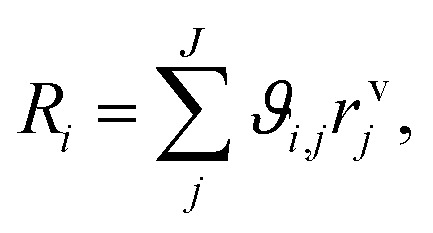
with

8 *r*^v^_*j*_ = (1 − *ε*_bed_)*ρ*_cat,*j*_*ξ*_cat,*j*_*r*^m^_*j*_.

In the above equations, *r*^v^_*j*_ and *r*^m^_*j*_ are the volume and mass specific rates of reaction *j* in mol m^3^ s^−1^ and mol kg^−1^ s^−1^, *ε*_bed_ is the porosity of the catalyst bed estimated to be 0.39, *ρ*_cat,*j*_ is the density of the catalyst that promotes reaction *j*, *i.e.*, the densities of the CZA and the γ-Al_2_O_3_ catalysts with the respective values of 1761.3 kg m^−3^ and 667.9 kg m^−3^, and *J* is the number of reactions. Finally, *ξ*_cat,*j*_ stands for the volume fraction of the catalyst that promotes reaction *j* calculated by,9
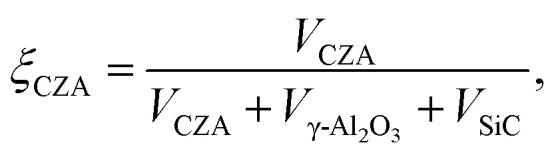
10
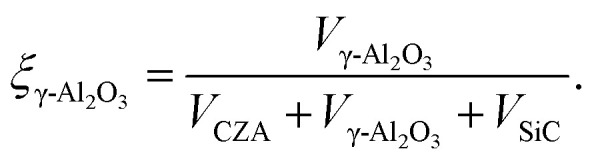
where *V*_CZA_, *V*_γ-Al_2_O_3__ and *V*_SiC_ are the volumes of CZA, γ-Al_2_O_3_ and SiC respectively.

### 3.2. Reaction kinetic model

For the initial model discrimination, the available experimental data were simulated using eight different kinetic models from the open literature.^15,21,22,30,31,39–41^ Subsequently, the five models with the lower residual squared sum were parameterized to fit the data. Our previous model^21^ exhibited the best agreement with the experimental data acquired for this contribution, which can be attributed to similar operating conditions, and to the fact that in both contributions the same catalysts (same supplier), and pre-treatment procedures were employed. The mechanistic assumptions and model structure were chosen for fine-tuning, and the model structure that enabled the best fit is presented in the following. Further information on the initial model discrimination is presented in the ESI,† along with a compilation of the rate expressions and specific parameters of the tested models (Table S2†). The new estimated model parameters are presented in Section 4.2.1 followed by the statistical evaluation of the estimates.

The reaction network considered in this model consists of the CO_2_ hydrogenation (reaction 1), the methanol dehydration to DME (reaction 2), and the water gas shift reaction (reaction 3). Reactions 1 and 3 are assumed to be promoted by the CZA catalyst, while reaction 2 is promoted by γ-Al_2_O_3_.

Reaction 1:CO_2_ + 3 H_2_ ⇌ CH_3_OH + H_2_O

Reaction 2:2 CH_3_OH ⇌ CH_3_OCH_3_ + H_2_O

Reaction 3:CO + H_2_O ⇌ CO_2_ + H_2_

The reaction rate expressions were postulated based on the general Hougen–Watson formulation,11
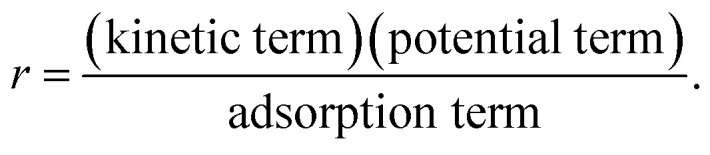


The kinetic term equals the rate constant of each reaction *j* (*k*_*j*_). The potential term, describing the driving force of the reaction *i.e.*, the distance from thermodynamic equilibrium, is defined for each reaction *j* as follows,12
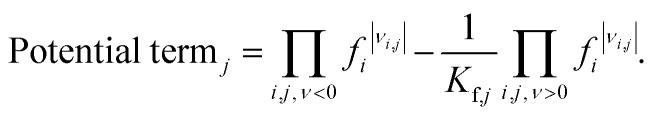


The adsorption term is generally defined by,13
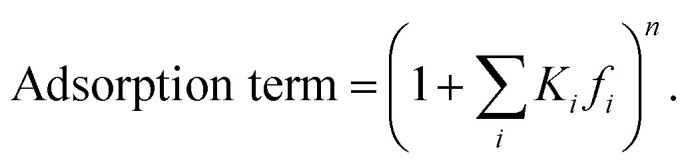


It accounts for the inhibition caused by adsorbed species on the catalytically active surface, and hence it must be defined for each component of the catalyst mixture. The postulated model includes the adsorption of CO_2_, CO and dissociated H_2_ on the CZA (eqn (14)), whereas no adsorption on the dehydration catalyst was considered (eqn (15)). Furthermore, the adsorption term has a different influence on the rates of the CO_2_ hydrogenation and the WGSR, with *n* = 3 and 1 respectively.^21,29^ In eqn (12) and (13), *f*_*i*_ is the fugacity of component *i* in bar, *K*_f,*j*_ is the equilibrium constant of the same reaction, *ν*_*i*,*j*_ is the stoichiometric coefficient of component *i* in reaction *j*, and *K*_*i*_ is the adsorption constant of component *i*.14
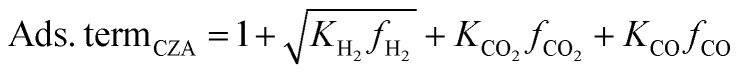

15 Ads. term_γ-Al2O3_ = 1

The resulting rate expressions for the three reactions are presented in eqn (16)–(18).16
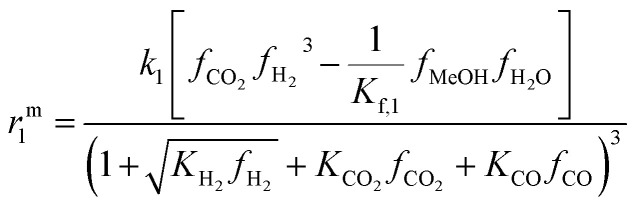
17
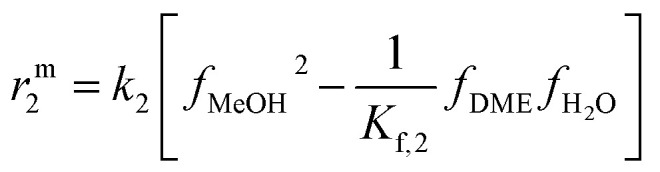
18
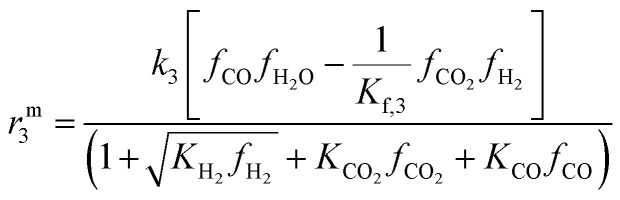


The reaction rate and adsorption constants (*k*_*j*_ and *K*_*i*_) are each calculated using modified Arrhenius and the van't Hoff equations (eqn (19) and (20)). This re-parameterization reduces the correlation between the frequency factor and the activation energy, as well as between the sticking coefficients and the enthalpy of adsorption.^42^ Other advantages of using re-parameterized expressions are lower computational costs and higher robustness in parameter estimation with the least squares algorithm.^43^ These are particularly relevant for the fitting of large data sets, as used in this work.19
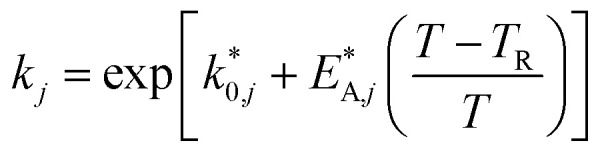
20
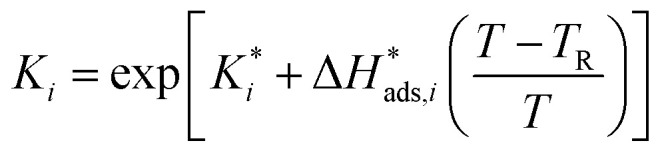


The modified parameters are related to the parameters of the traditional Arrhenius equation according to eqn (21) and (22).^34^21
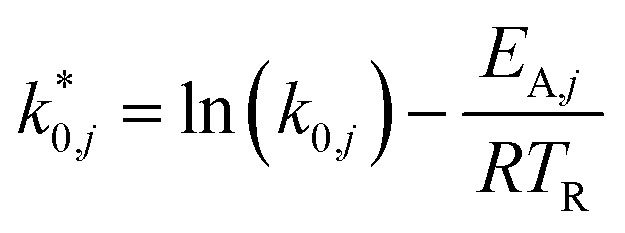
22
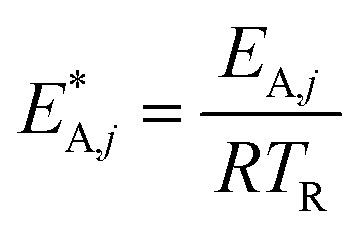
the same applies to the van't Hoff equation as follows,23
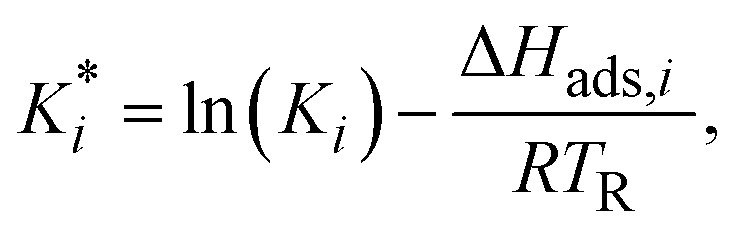
24
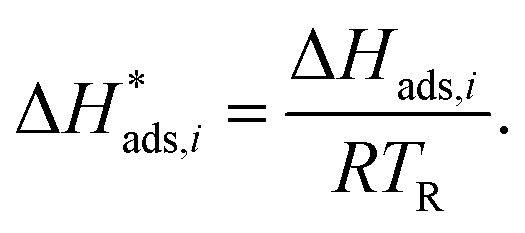


The reference temperature (*T*_R_) was calculated with eqn (25) based on the temperature of each experiment *n*.^44^25
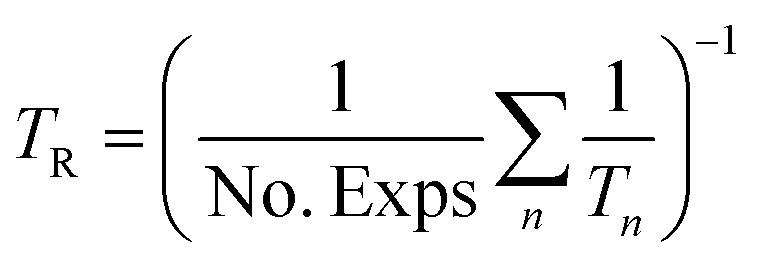


The equilibrium constants *K*_f,*j*_ of each reaction *j* are calculated using eqn (26),^45^ the temperature *T* in K, and the parameters in Table 3.26
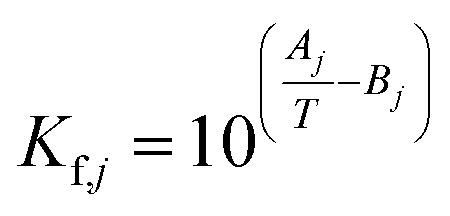


**Table:** Parameters for the calculation of eqn (26).^21^

Parameter	Reaction 1	Reaction 2	Reaction 3
*A*	3014.4029	1143.9494	2076.2131
*B*	10.3856	0.9925	2.0101

The equilibrium constants are dimensionless for reactions 2 and 3 (methanol dehydration to DME, and WGSR), while *K*_f,1_ (the equilibrium constant of CO_2_ hydrogenation to methanol) has the units bar^−2^, in accordance with the law of mass action.

For performance evaluation, the conversion of component *i* (*X*_*i*_), and the carbon-normalized yield and selectivity of component *i* from CO_*x*_ (*Y*_*i*_ and *S*_*i*_) were computed based using eqn (27)–(29), respectively.27
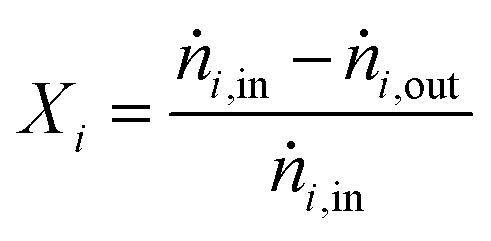
28
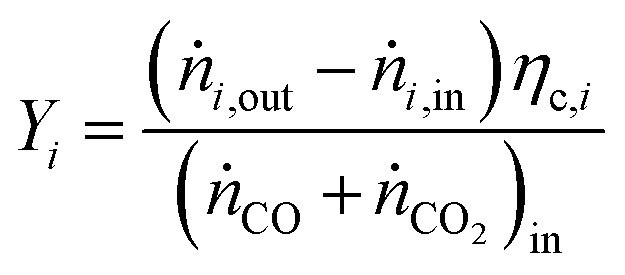
29
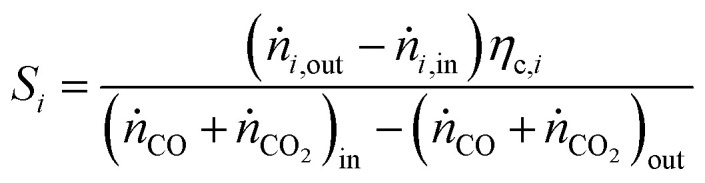
In these equations, *ṅ*_*i*_ is the molar flow of component *i*, *η*_c,*i*_ is the number of carbon atoms in the same component, and the subscripts “in” and “out” refer to the respective quantities at the reactor inlet and outlet.

## 4. Results and discussion

In this section, experimental results will be presented (Section 4.1), followed by the modelling results and mechanistic analysis (Section 4.2). Since most of the studies for the direct DME synthesis have been carried out with a catalyst weight ratio of one (*μ* = 1), this catalyst ratio is treated here as the reference composition for the evaluation of experimental and simulations results. The results are presented for the highest space-time (at which the effects are more pronounced) unless otherwise stated.

### 4.1. Experimental results

This section presents an overview of the effects observed experimentally. To determine causality and for a comprehensive understanding of the phenomena, the reactions kinetics are studied and analyzed in Section 4.2 in the light of the derived kinetic model and further kinetic studies from the literature.

**Fig. 1:**
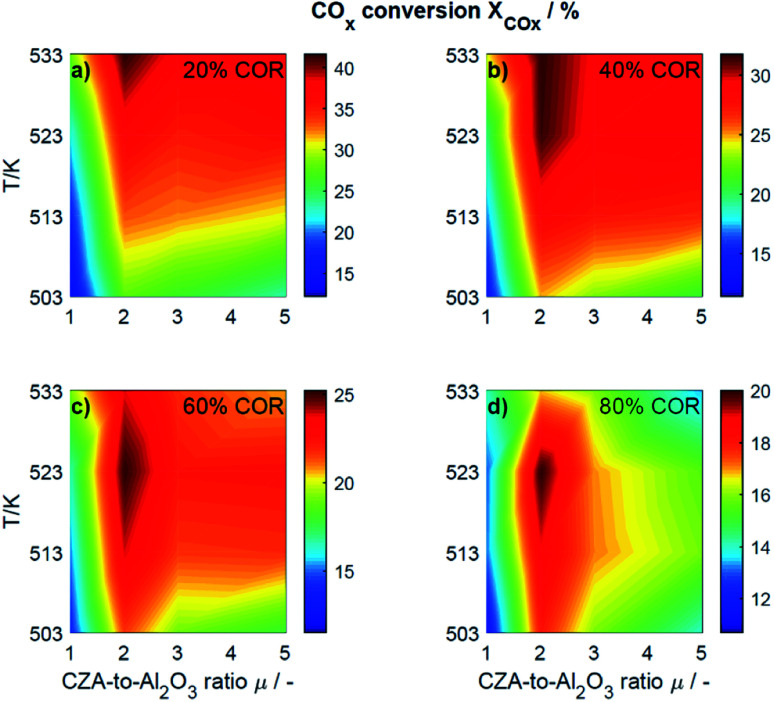
Conversion of CO_*x*_ determined experimentally and plotted as a function of the temperature (*T*) and the CZA-to- γ-Al_2_O_3_ ratio (*μ*) for nominal CORs of (a) 20%, (b) 40%, (c) 60% and (d) 80%. Experimental conditions summarized in Table 2.

**Fig. 2:**
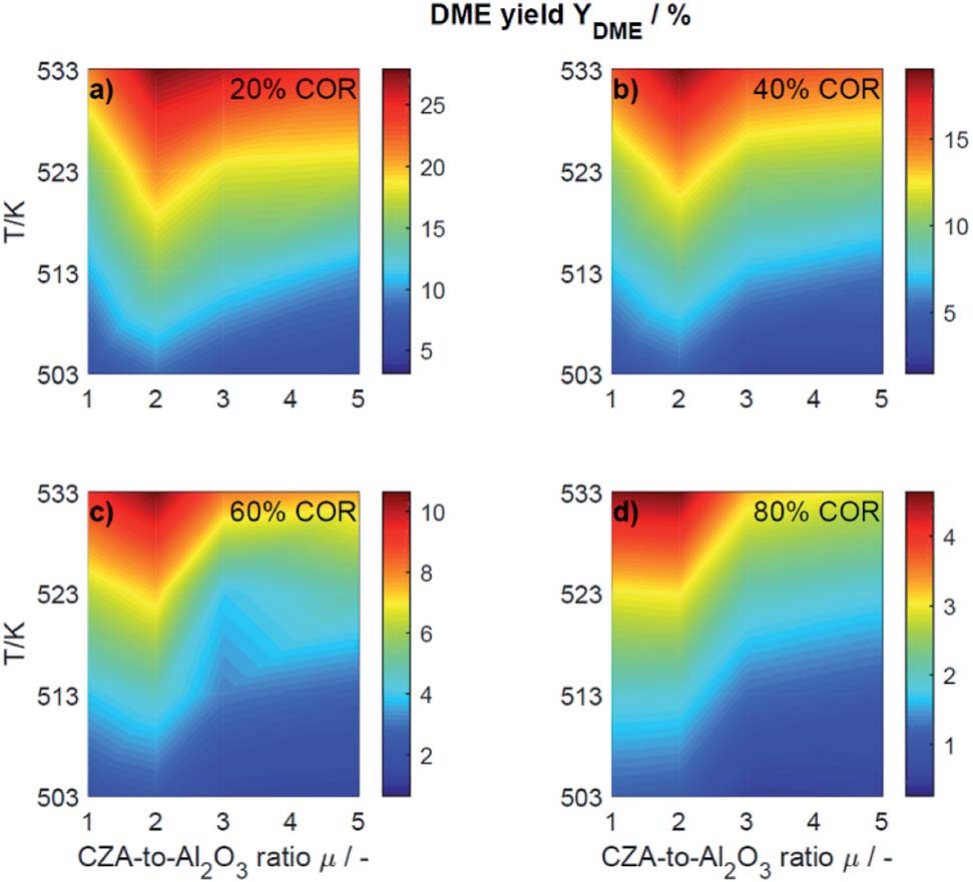
Yield of DME determined experimentally and plotted as a function of the temperature (*T*) and the CZA-to- γ-Al_2_O_3_ ratio (*μ*) for nominal CORs of (a) 20%, (b) 40%, (c) 60% and (d) 80%. Experimental conditions summarized in Table 2.

For an initial qualitative analysis of the experimental results, the measured conversion of CO_*x*_ (*X*_CO_*x*__) and DME yield (*Y*_DME_) are shown in Fig. 1 and 2 as a function of the temperature and the CZA-to-γ-Al_2_O_3_ ratio (*μ*) for the four investigated COR levels (20, 40, 60 and 80%). To create this graphical representation, the values between the experiments were calculated using lowpass interpolation with the Matlab® function *interp*. The maximal conversion attained for the different inlet feed composition varies from 19.8% (COR = 80%, *T* = 523 K, *μ* = 2) to 42.6% (COR = 20%, *T* = 533 K, *μ* = 2). In general, low CORs, *i.e.*, low CO_2_ contents in the feed, lead to higher conversions at all temperatures. The highest conversions were reached in all cases with *μ* = 2, whereas the conversions attained with the reference catalyst bed composition (*μ* = 1) are the lowest. Even at high temperatures relatively low conversions are attained with the reference *μ* = 1 in comparison to those reached with the other catalyst beds. It is obvious that the temperature at which the maximal conversion was measured, decreases with increasing CORs.

The DME yield, displayed in Fig. 2, exhibits a strong temperature dependency. The maximal *Y*_DME_ varies between 4.6% (COR = 80%, *T* = 533 K, *μ* = 2) and 27.9% (COR = 20%, *T* = 533 K, *μ* = 2). Overall, lower CORs lead to higher yields of DME, and analogous to the conversion of CO_*x*_, the highest yields were attained with a CZA-to-γ-Al_2_O_3_ ratio *μ* = 2. The response surfaces are very similar for all CORs, however, it can be observed that with increasing COR, the region at which the highest yields are reached migrates towards the upper left corner *i.e.*, towards high temperatures and low *μ*. At 533 K and 20% COR for example, high yields are attained with all the catalyst beds, whereas at 80% COR, the yields reached at this temperature are high with *μ* up to two, and significantly lower with *μ* of three and higher.

**Fig. 3:**
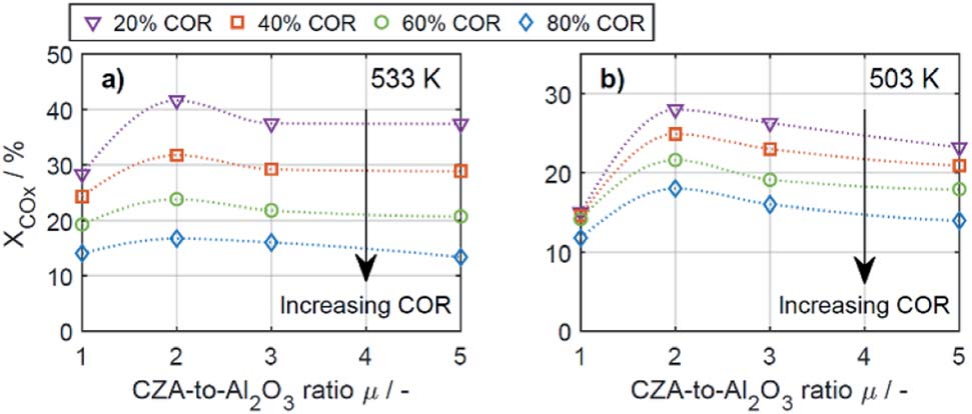
CO_*x*_ conversion as a function of the CZA-to-γ-Al_2_O_3_ ratio (*μ*) for CORs from 20% to 80%. (a) *T* = 533 K and (b) *T* = 503 K.

To enable a quantitative analysis of the observed effects, representative results at the minimal and maximal temperature are investigated more in detail in the following. The CO_*x*_ conversion is depicted in Fig. 3 for the investigated CORs as a function of the CZA-to-γ-Al_2_O_3_ ratio, at the maximal and minimal temperature of 533 K and 503 K (Fig. 3a and b). At 533 K, the CO_*x*_ conversion increased for all measured feeds when increasing *μ* up to a value of 2. This effect was most pronounced for a COR of 20% where the relative enhancement of the conversion was of 47%. For a COR of 80% the relative enhancement amounted 19%. A further increase of the CZA-to-γ-Al_2_O_3_ ratio had a negative effect on the conversion compared to the conversion obtained with *μ* = 2, but in all cases, the attained values were still higher than in the reference case (*μ* = 1). The only exception to this observation was for COR = 80% and *μ* = 5, where the conversion decreases from 14% (*μ* = 1) to 13% (*μ* = 5).

At a temperature of 503 K, the conversion of CO_*x*_ shown in Fig. 3b for all bed compositions and CORs is lower than for the corresponding values attained at 533 K, which can attributed to the general dependency of the reaction rates on the temperature. For all CORs, a maximum at *μ* = 2 was detected. With this CZA-to-γ-Al_2_O_3_ ratio, a relative conversion enhancement of 88% and 52% was obtained compared to *μ* = 1 at the minimal and maximal COR levels of 20% and 80% respectively. Comparable to the observations made at 533 K, the effect of the catalyst bed composition on the conversion is more pronounced at lower CORs. Furthermore, it can be observed that with the reference catalyst ratio *μ* = 1, the attained CO_*x*_ conversion is at a close value of approx. 14% regardless of the CO_2_ content in the inlet feed, in contrast to the other experiments with increasing CO_*x*_ conversion as the COR is decreased.

In general it was observed that decreasing amounts of CO_2_ in the feed gas (*i.e.*, decreasing CORs) lead to higher conversions, and to more pronounced effects of the catalyst bed composition. The beneficial effect of low CO_2_ concentration in the synthesis gas has been observed in other kinetic studies of both the methanol and the DME synthesis.^14,15,26,46–48^ Regarding the surface chemistry, low CO_2_ concentration prevents sintering of the CZA catalyst, and promotes catalyst morphology that enhances the catalytic activity.^48,49^ From a thermodynamic perspective, high CO_2_ feed concentration shifts the equilibrium of the WGSR towards the educts (H_2_O and CO), resulting in increased water formation and subsequently in decrease of the methanol dehydration rate.^14,15^ This explanation is in accordance with our findings and is further confirmed by increased methanol selectivity at high CORs discussed in the following. In addition, we explain this effect on the basis of mechanistic considerations in Section 4.2.2.1.

**Fig. 4:**
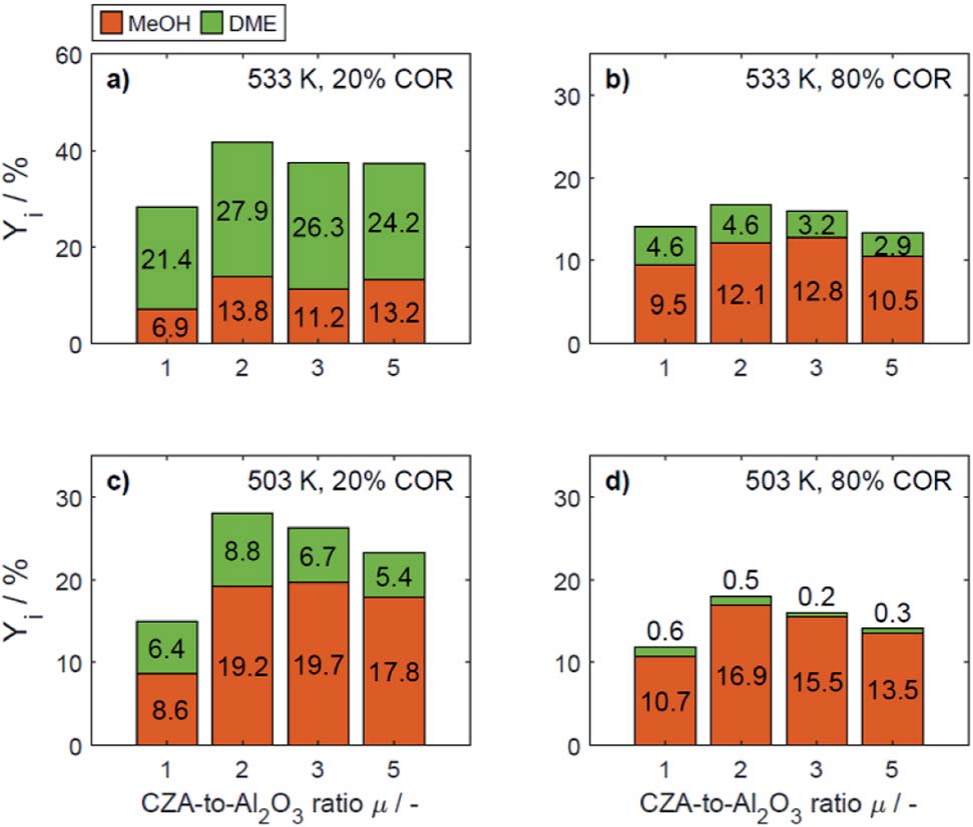
Yield of methanol and DME at specific conditions: (a) 533 K, 20% COR, (b) 533 K, 80% COR, (c) 492 K, 20% COR and (d) 492 K, 80% COR.

In Fig. 4a–d the yields are shown for the minimal and maximal CORs 20% and 80%, and for the minimal and maximal temperatures 503 K and 533 K. Since the yield is calculated based on the reacted CO_*x*_, and no other carbon-containing compounds were detected in a significant amount during the experiments, the yield is calculated only for methanol and DME. However, as discussed further in Section 4.2, CO and CO_2_ formation was evidenced at some specific conditions.

At 533 K and a COR of 20% (Fig. 4a), the converted CO_*x*_ in the feed gas reacted to form mainly DME. In general, at this temperature and COR, an increased amount of the CZA catalyst led to a higher DME production than that attained with the reference catalyst bed (*μ* = 1). The highest relative enhancement of the DME yield was 30.3% with *μ* = 2. A further increase of *μ* = 3 and 5 also enhanced the yield of DME but to a lower extent (enhancement of 22.8% and 13.2% respectively compared to the yield attained with the reference *μ* = 1). At the same temperature and a COR of 80% (Fig. 4b), the methanol yield was at least twice as high as that of DME. An increased *μ* did not increase the DME yield which amounts 4.6% at *μ* = 1 and 2, and was lower otherwise. Comparing the results shown in Fig. 4a and b (and also Fig. S3a and b†), a shift of the selectivity from DME to methanol is observed when increasing the COR from 20 to 80%. The water concentration is low at high CO contents in the feed (water removal *via* WGSR), and high at a high level of CO_2_.^14^ Obviously, presence of water is thermodynamically unfavorable for the dehydration, explaining the observed methanol concentration at high CORs. This conclusion is supported by the mechanistic analysis provided in Section 4.2.2.1.

In Fig. 4c and d it is observable that for a temperature of 503 K, the yield of methanol is higher than that of DME for both COR levels. An enhancement of the DME yield compared to the reference case is still observable at a COR of 20% (38.2% and 4.3% with *μ* = 2 and 3), whereas at 80% COR, an increase of the *μ* proved to be disadvantageous for the DME yield. The lowest DME yields were observed at 503 K, a COR of 80% and *μ* = 3 and 5.

The catalytic activity of the CZA/γ-Al_2_O_3_ system is a function of combined physicochemical characteristics such as Cu surface area, dispersion, and acidity.^50–52^ Furthermore, the setup of reaction conditions have also shown to be a key factor.^24^ While the study of the catalysts properties was out of the scope of this work, a wide range of conditions was covered during the experimental program. The improvement observed by increasing the CZA-to-γ-Al_2_O_3_ ratio reveals that the number of required acid sites has already been significantly exceeded when equivalent catalysts masses are used.^15,21^ Therefore, an increase of the catalyst ratio leads to an overall enhancement of the synergetic effects of the direct DME synthesis *i.e.*, the faster methanol formation due to an increased amount of CZA catalyst has a positive effect on the methanol dehydration even though the amount of the catalyst that promotes this reaction is reduced. Overall, it was observed that the highest enhancement of the DME yield was attained at a CZA-to-γ-Al_2_O_3_ ratio of *μ* = 2, and that higher ratios lead to a minor improvement, or even to a decrease of the DME production. Additionally, it was observed that the methanol yield increased with increasing CZA-to-γ-Al_2_O_3_ ratio at all conditions (Fig. 4a–d) as also described in other kinetic studies.^15,24,50^ Hence, the evidenced enhancement of the DME yield is associated to the higher conversion, *i.e.*, the conversion of CO_*x*_ increased more than the DME selectivity decreased, leading to higher DME yields than with the reference catalyst bed.

### 4.2. Modeling results

Predictive models able to make accurate predictions over a wide range of conditions are of considerable importance as a basis for model-based optimization and for the design of novel reactor concepts. The respective contribution of our work is a reaction kinetic model for direct DME synthesis suitable these purposes. In Section 4.2.1, the results of the parameter estimation are presented together with an analysis of the achieved goodness of fit and statistical significance of the parameter estimates. In Section 4.2.2, the phenomena experimentally observed (Section 4.1) are explained taking into account the derived kinetic model. In addition, we describe to what extent our findings are consistent with the results and new mechanistic insights of other studies.

#### 4.2.1. Reaction kinetic model

**Fig. 5:**
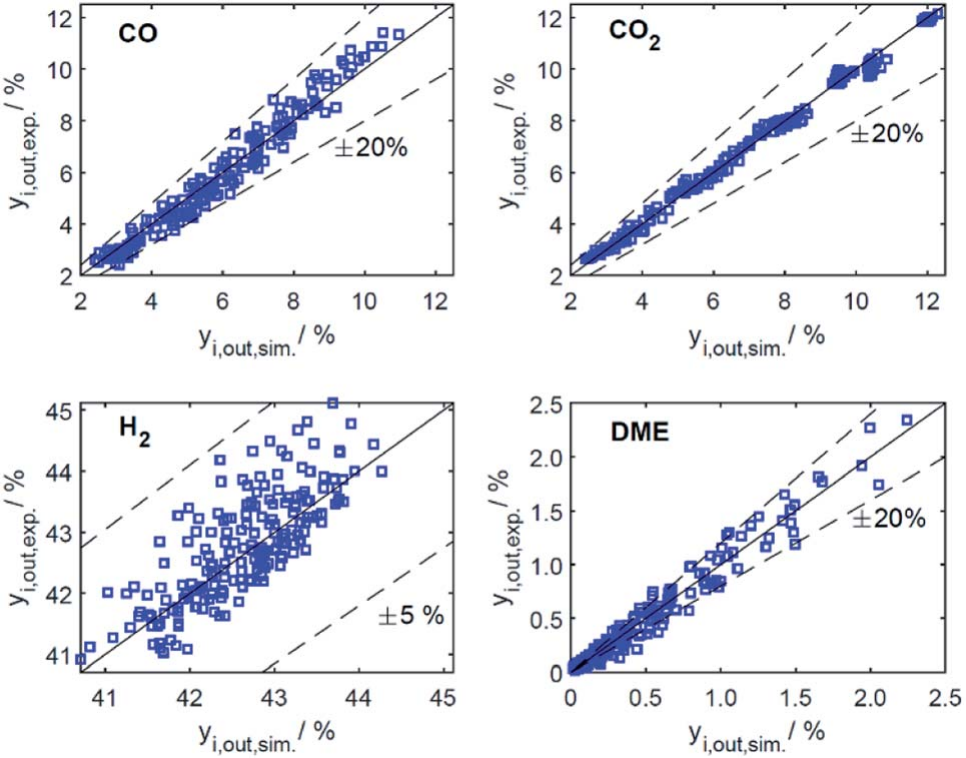
Parity plots for mole percent of CO, CO_2_, H_2_ and DME in the product gas for all data (186 experiments).

In this section, the resulting kinetic model, *i.e.*, the parameter estimates and model evaluation are discussed. As mentioned briefly in Section 3.2, the presented model was the one that enabled the best fit of the experimental data within the entire range of conditions investigated in this work. The derived model chosen after a discrimination procedure agrees with the one derived from mechanistic assumptions by Lu *et al.*^29^ and used in a previous work.^21^ It considers the linearly independent reactions CO_2_ hydrogenation and WGSR, along with the methanol dehydration to DME. In agreement with the mentioned studies, including no adsorption term for the dehydration catalyst, and the adsorption of CO, CO_2_ and dissociated H_2_ on the CZA catalyst led to the best representation of the experimental data. Considering the adsorption of water and methanol as done in other kinetic studies of the direct DME synthesis^15,30,53^ worsen the quality of fit, and was therefore discounted from the model structure. The goodness of fit for CO, CO_2_, H_2_ and DME with the resulting model is represented by the parity diagrams in Fig. 5 with the measured quantities plotted against the numerically predicted ones. The model-specific parameters were estimated based on 186 experimental data points. The mean relative error between the predicted and measured molar fractions over all data amount to 2.7% for CO_2_, 7.2% for CO, 1.0% for H_2_, and 22.3% for DME. The deviation of the DME predictions is mostly attributed to an over-prediction of the data measured with Mu = 5. The data taken with this catalyst bed exhibits the lowest DME production and low DME mole fractions in the product gas as shown in Fig. 2. Hence, these measurements have a high signal-to-noise ratio, and a lower measurement accuracy, to which the larger deviations can be attributed to. Nonetheless, the deviation of the DME predictions is considered acceptable, especially regarding the extensive range in which the experiments were measured. Furthermore, the predictions lie with a clear tendency and a weak scattering along the bisector (*y* = *x*), and no systematic deviations are identifiable for any of the species.

The resulting parameter estimates are shown in Table 4 along with the respective 95% confidence intervals. The adsorption parameters were fixed. Hence, no statistical information is available on these estimates. In regards to the rate constants, the confidence intervals demonstrate that all re-parameterized pre-exponential factors and activation energies are statistically significant. Moreover, the width of the confidence intervals is less than 13% of the respective estimates for five out of six parameters. The widest confidence interval was that of the re-parameterized activation energy of the CO_2_ hydrogenation, with a width of 29% of the estimated value, which underlines the high statistical significance of the estimated kinetic parameters.

Estimated parameters in re-parameterized form according to eqn (19) and (20), and 95% confidence intervals

**Table:** 

Reaction	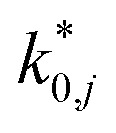	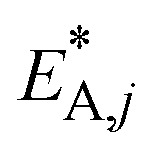
CO_2_ hydrogenation	3.19 (±0.04) mol kg^−1^ s^−1^ bar^−4^	7.60 (±2.20)
MeOH dehydration	−5.72 (±0.07) mol kg^−1^ s^−1^ bar^−2^	24.58 (±3.22)
WGSR	1.74 (±0.11) mol kg^−1^ s^−1^ bar^−2^	40.77 (±4.96)

**Table:** 

Adsorbate	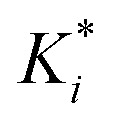	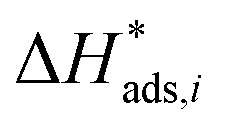
CO_2_	4.68 bar^−1^	−1.25
CO	−34.04 bar^−1^	−79.81
H_2_	7.13 bar^−1^	−5.04

The reference temperature was calculated as *T*_R_ = 517.43 K using eqn (25) for the 186 experiments used for fitting.

Notice that 
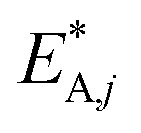
 and 
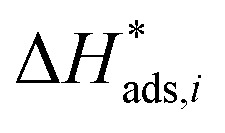
 are dimensionless according to eqn (22) and (24), and that 
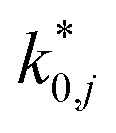
 is based on the mass of the catalyst that promotes each reaction, *i.e.*, CZA for the CO_2_ hydrogenation and the WGSR, and γ-Al_2_O_3_ for the methanol dehydration to DME.

The adsorption constants *K*_*i*_ were calculated with eqn (20) at the different temperature levels to determine the influence of the adsorption of each species on the adsorption term (the reported value for H_2_ corresponds to 
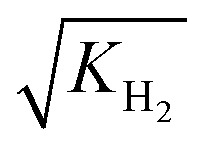
 according to eqn (14)). The calculated values are shown in Table 5. The CO adsorption has clearly the lowest adsorption constant, in agreement with the studies of Lu *et al.*^29^ and Delgado Otalvaro *et al.*^21^ where the same adsorption term was employed. The constant of CO_2_ adsorption exhibited both in Delgado Otalvaro *et al.*^21^ and in the present work the highest value. This is also consistent with the investigations of Klier *et al.*,^47^ where a strong CO_2_ adsorption on the metallic catalyst was observed. All adsorption constants shown in Table 5 decrease with increasing temperature due to the exothermal nature of adsorption.

**Table:** Adsorption constants at different temperatures

	*T* = 503 K	*T* = 513 K	*T* = 523 K	*T* = 533 K
*K*_CO_2__/bar^−1^	111.9	109.2	106.6	104.1
*K*_CO_/bar^−1^	1.6 × 10^−14^	3.2 × 10^−15^	6.9 × 10^−16^	1.6 × 10^−16^
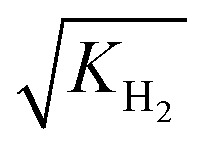 /bar^−0.5^	37.9	36.0	34.3	32.8

Binary correlation coefficients (*ρ*_*i*,*j*_) were computed to assess the correlation between the parameter estimates (Table 6). The absolute values of all the non-trivial correlations coefficients confirm that using the re-parameterized Arrhenius and van't Hoff equations (eqn (19) and (20)) led successfully to a weak correlation between the parameter estimates. In addition, the convergence time of the fitting was reduced by about 60% after applying re-parameterization.

**Table:** Binary correlation coefficients of parameter estimates

*ρ*_*i*,*j*_	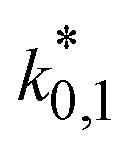	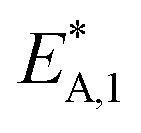	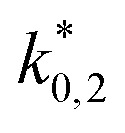	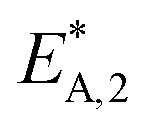	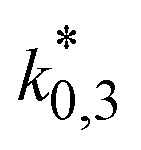	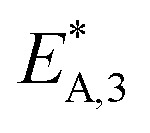
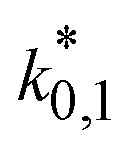	1	−0.53	−0.83	0.40	−0.39	0.28
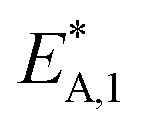		1	0.38	−0.85	0.31	−0.36
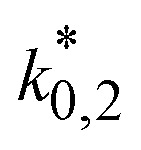			1	−0.44	−0.07	−0.11
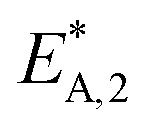				1	−0.11	−0.03
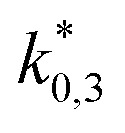					1	−0.28
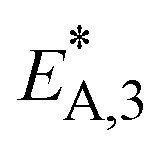						1

#### 4.2.2. Mechanistic analysis

Using the derived model, the proposed reaction mechanism is elucidated in the following based on representative results. The influence of the COR, the temperature, and the CZA-to-γ-Al_2_O_3_ ratio on the reactions rates is discussed, as well as the observed CO and CO_2_ formation during reaction.

##### 4.2.2.1 Effect of the COR

In Section 4.1 it has been shown that high conversions and yields of DME are attained at the lowest COR levels. This was observed at all conditions in the investigated operating range, although at differing extent. This is in accordance with former kinetic studies of the methanol,^26,47,48^ and DME synthesis^14,15^ which have shown that an optimal CO_2_ feed concentration exists, at which both the methanol formation and subsequently the DME formation are favored, while exceeding this concentration leads to reduced conversions and yields. Sintering of Cu crystallites in the CZA catalyst takes place with CO/H_2_ and CO_2_/H_2_ feeds due to Cu segregation from ZnO, and due to the presence of water respectively. However, sintering is prevented at the optimal CO_2_ feed concentration.^48^ Since we observed no optimal value for the COR within the investigated operating range, we conclude, in agreement with other studies,^14,15,47,48^ that the optimal value is probably less than or equal to 3%, which was the lowest CO_2_ concentration considered in this work (at 20% COR).

**Fig. 6:**
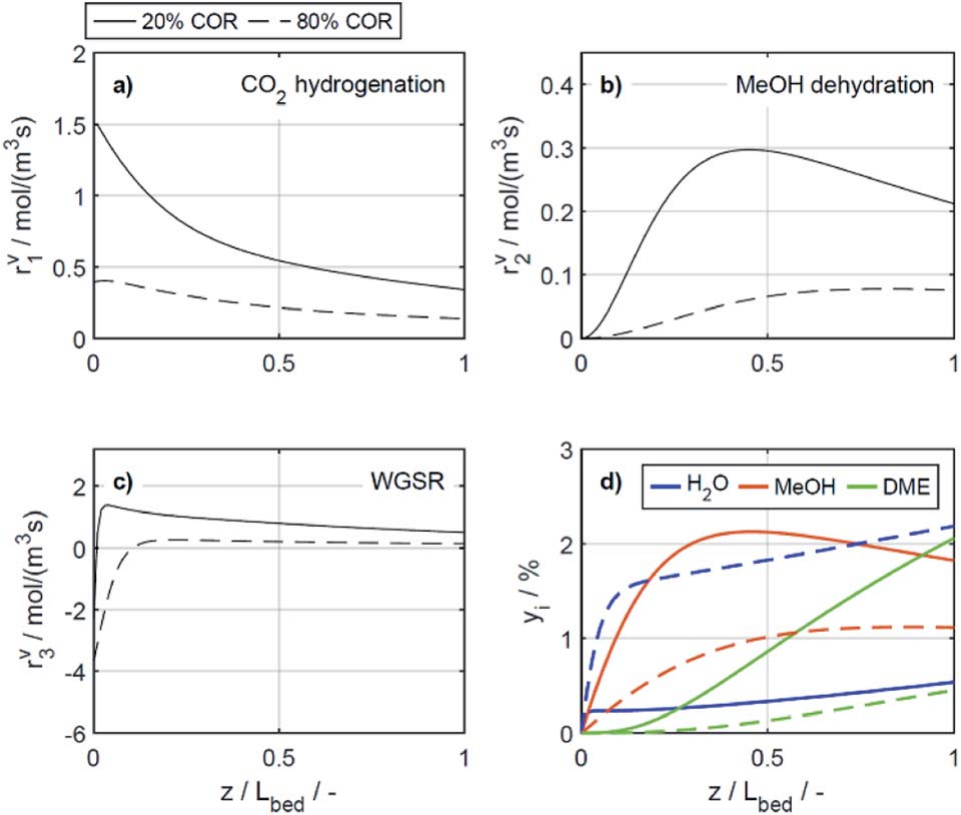
Reaction rates (a) CO_2_ hydrogenation, (b) MeOH dehydration, (c) WGSR and (d) mole percentage profiles of water, methanol and DME at *T* = 533 K, *μ* = 2. (—) Solid lines: 20% COR, (- - -) dashed lines: 80% COR.

To elucidate the effect of the COR on the reactions rates, these have been depicted in Fig. 6a–c at exemplary conditions for the minimal and maximal CORs of 20% and 80%. Additionally, the mole percentage profiles of water, methanol and DME are displayed in Fig. 6d (Fig. S4 in the ESI† includes the profiles of CO and CO_2_, which were left out here for better visualisation). It is shown that the rates of the three reactions, *i.e.*, CO_2_ hydrogenation, methanol dehydration and WGSR, are higher at 20% COR than at 80% COR. This effect is straight forward for the WGSR where CO_2_ is a product, and an increased product concentration shifts the equilibrium towards the educts according to the Le Chatelier's principle. For the CO_2_ hydrogenation on the other hand, it may appear contradictory that the rate is lower at higher CORs since CO_2_ is a reactant in this reaction. This has been attributed to several factors in the literature such as to the presence of water in high concentrations leading to sintering of the Cu particles,^48^ to thermodynamic limitation of the methanol formation,^54^ or to strong CO_2_ adsorption on the metallic catalyst.^47^ CO_2_ adsorption is also believed to be important in our study, which is accounted for in the model by the strong influence of CO_2_ concentration on the adsorption term (eqn (14) and Section 4.2.1), and by the considerable influence of the adsorption term on the CO_2_ hydrogenation (eqn (16)). The strong influence of the adsorption term leads to an overall decrease of the reaction rate with increasing CO_2_ in the feed, even though the potential term of the forward reaction is indeed higher at higher CORs.

The rate of the WGSR (Fig. 6c) takes on negative values at the reactor entrance at both CORs, indicating that the reverse water gas shift reaction (rWGSR) is faster than the WGSR at the inlet conditions. At 80% COR the rWGSR is particularly fast (high negative values, min. *r*^v^_3_ = −3.7 mol m^3^ s^−1^ at *z* = 0), which we attribute to the high concentrations of CO_2_ and H_2_ in the feed. Although a high hydrogen feed concentration is necessary to avoid the stoichiometric limitation of CO_2_ hydrogenation to methanol, the high feed concentration of both, CO_2_ and H_2_, accelerates the rWGSR instead of the CO_2_ hydrogenation as evidenced, leading to water and CO production.^16,55^ The simulations show that the rWGSR prevails over the WGSR for the initial 10% bed length, resulting in a pronounced increase of water concentration (Fig. 6d, blue dashed line). From a bed length beyond 10%, the water gas shift equilibrium (reaction 3) shifts to the right side and *r*^v^_3_ takes on nearly constant positive values over the entire following bed length, accompanied by reduced overall water formation as water is partially consumed by the WGSR. The widely accepted mechanism of methanol formation by CO_2_ hydrogenation over copper-based catalysts was disputed by Gaikwad *et al.*^56^ It was shown by means of space-resolved experiments that the main carbon source for methanol formation from CO_2_ rich feeds depends on the reaction conditions, in particular on the temperature. The authors concluded that at 533 K and CO_2_/H_2_ feeds, methanol formation takes place *via* CO hydrogenation formed by the rWGSR at the reactor inlet. Our simulation results are in accordance with that conclusion, *i.e.*, the rWGSR takes place at the reactor inlet, followed by the CO hydrogenation, in the model described by the WGSR and the subsequent CO_2_ hydrogenation. We also believe that this explains the higher conversions and yields at high CO feed concentration. At this COR, the rWGSR prevails only at the reactor entrance (up to 0.8% reactor length), and the rate does not reach such high negative values (min. *r*^v^_3_ = −2.2 mol m^3^ s^−1^ at *z* = 0). As a result, the water concentration at the reactor entrance rises steeply, but does not reach such a high level as at 80% COR. Although water has shown to limit the catalyst deactivation by coke deposition^31,57^ high water concentration in is indisputably detrimental for direct DME synthesis, especially when using γ-Al_2_O_3_ as the dehydration component.^4,54^ This underlines the importance of water removal, *e.g.*, by permselective membranes^58,59^ which could also be axially tailored to counteract the observed steep water increase at the reactor entrance shown here as well as in other kinetic studies.^60,61^

Clearly, the methanol dehydration to DME is also affected strongly by the COR, as shown in Fig. 6b. At 20% COR the concentration of methanol is higher than the concentration of water for the largest portion of the reactor (solid lines in Fig. 6d). Conversely, at 80% COR the water concentration is higher than the concentration of methanol (dashed lines in Fig. 6d). Reduced methanol dehydration rate at high CORs has been explained in the literature by deactivation phenomena of the γ-Al_2_O_3_, and by a strong adsorption of methanol and/or water on the surface of the dehydration catalyst.^14,28,30,62^ In our experiments, no activity drop was observed and, as mentioned in Section 3.1, the model that enabled the best fit to the experimental data is based on the assumption that no adsorption on the dehydration catalyst takes place.^21,29,54^ Hence, the influence of the COR on the dehydration rate is accounted for by thermodynamics only. *I.e.*, considering the stoichiometry of the dehydration reaction it is clear that high methanol and low water concentrations as evidenced at 20% COR are thermodynamically favorable for DME formation, while low methanol and high water concentrations as exhibited at 80% COR are disadvantageous. As a result, the methanol dehydration is significantly slower at 80% COR than at 20% COR explaining the decreasing DME formation with increasing CORs observed experimentally (Fig. 2 and 4a, b).

##### 4.2.2.2 Effect of the temperature

**Fig. 7:**
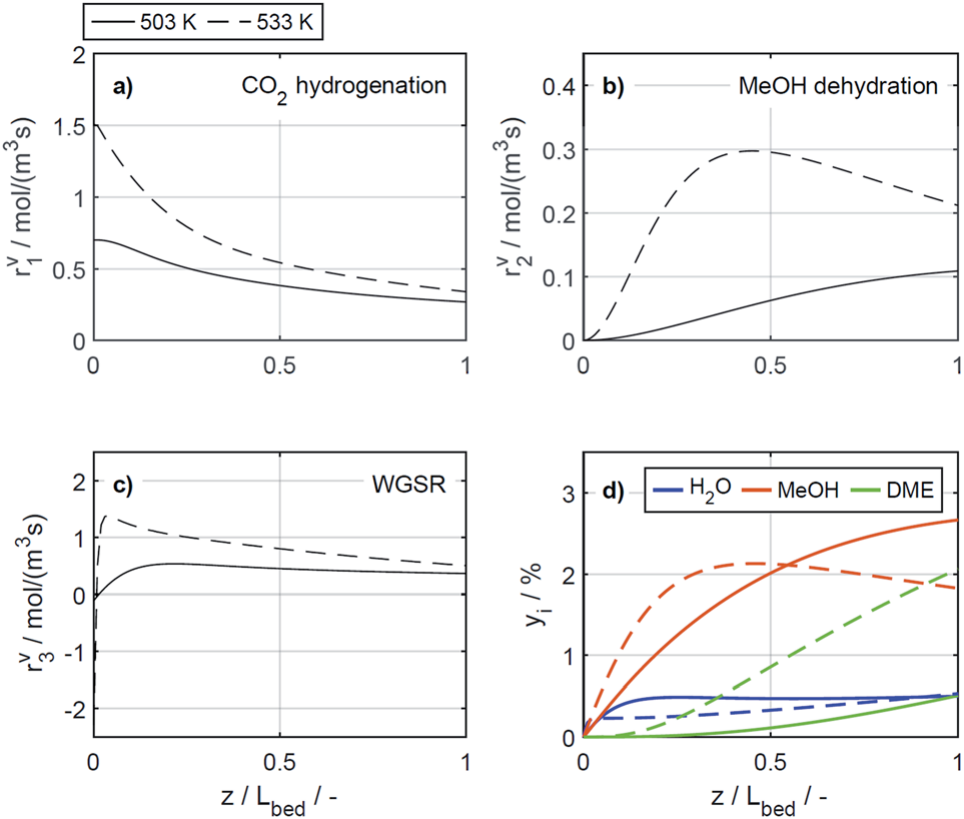
Reaction rates (a) CO_2_ hydrogenation, (b) MeOH dehydration, (c) WGSR and (d) mole percentage profiles of water, methanol and DME at *μ* = 2 and COR = 20%. (—) Solid lines: *T* = 503 K, (- - -) dashed lines: *T* = 533 K.

The reaction rates, and the mole fractions of DME, water and methanol are depicted in Fig. 7 at the minimal and maximal evaluated temperatures, *i.e.*, at 503 K and 533 K for a COR of 20%. Due to the general temperature dependence of the reaction rate constants, all reactions proceed faster at 533 K than at 503 K (Fig. 7a–c). In addition to the temperature dependence of the rate constants, the dependence of the adsorption rates is also relevant when assessing the influence of temperature based on the proposed model. Adsorption constants decrease with increasing temperatures due to the exothermal nature of adsorption processes (Table 5). Since the adsorption terms have an indirect proportional effect on the reaction rates (eqn (11)), the slower adsorption also contributes to the higher rates of the CO_2_ hydrogenation and WGSR evidenced at higher temperatures.

A factor not considered by the model but potentially favoring methanol dehydration at elevated temperature is enhanced water desorption from the dehydration catalyst surface,^63^ leading to an increased number of available active centres for the dehydration reaction. The effect of the temperature on the concentrations profiles is shown in Fig. 7d. Compared to 503 K (solid lines), at 533 K (dashed lines) the methanol concentration is higher for 55% of the reactor length, while the water concentration is lower for almost the entire reactor. Hence it is obvious that at 533 K, the driving force of the dehydration reaction is increased, leading to significantly higher DME concentrations and DME yields, as also determined experimentally (Fig. 4a and c). Furthermore, the concentration increase for DME is significantly higher than for methanol, confirming that higher temperatures have a positive effect on DME selectivity^24^ (Fig. S2 and S3†).

**Fig. 8:**
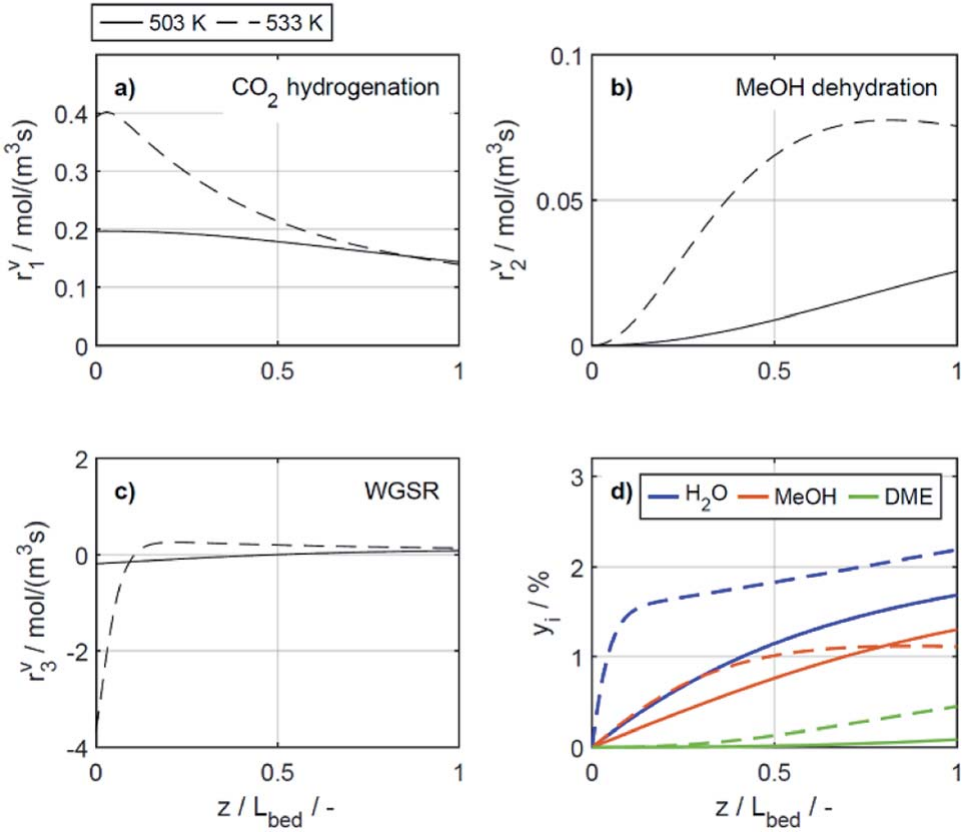
Reaction rates (a) CO_2_ hydrogenation, (b) MeOH dehydration, (c) WGSR and (d) mole percentage profiles of water, methanol and DME at *μ* = 2 and COR = 80%. (—) Solid lines: *T* = 503 K, (- - -) dashed lines: *T* = 533 K.

In the study of Gaikwad *et al.*,^56^ for methanol synthesis at 453, 533 and 613 K, the authors concluded that at 533 K the main reaction mechanism takes place *via* rWGSR and CO hydrogenation, while at lower temperature, direct CO_2_ hydrogenation is the dominant pathway. In Fig. 8, simulation results at the highest COR considered (80%) and at 503 and 533 K show that our lumped kinetic model is mechanistically sound according to these new insights. The respective reaction rates of the WGSR (Fig. 8c) are of particular interest: at 533 K, the phenomenon described in Section 4.2.2.1 takes place; *i.e.*, the rWGSR dominates at the reactor inlet, followed by both, WGSR and CO_2_ hydrogenation, in combination representing a descriptor for CO hydrogenation; at 503 K, the WGSR rate is nearly zero and shows a nearly constant profile along the reactor length. This leads us to the conclusion that at 503 K, methanol formation takes place *via* direct CO_2_ hydrogenation. From the findings of Gaikwad *et al.*,^56^ it cannot be concluded exactly at which temperature the mechanism shifts, although from our findings it seems plausible that at 503 K, both reaction pathways are contributing.

##### 4.2.2.3 Effect of the catalyst bed composition

**Fig. 9:**
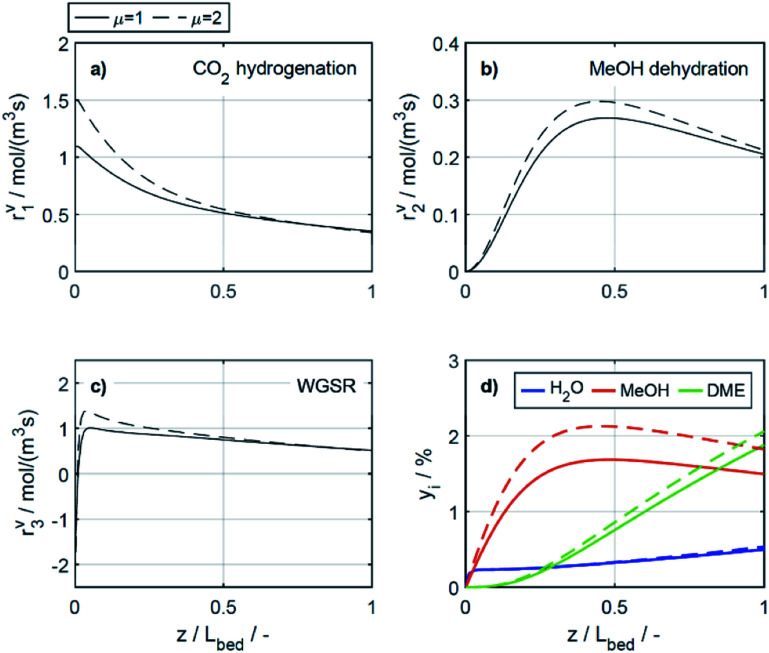
Reaction rates (a) CO_2_ hydrogenation, (b) MeOH dehydration, (c) WGSR and (d) mole percentage profiles of water, methanol and DME at *T* = 533 K, COR = 20%. (—) Solid lines: *μ* = 1, (- - -) dashed lines: *μ* = 2.

There are several studies concerning the catalyst bed composition for the direct DME synthesis. A literature overview recently provided by Peinado *et al.*^24^ summarizes that most studies have been performed for CO_2_ lean feeds and, with high CZA proportions in the catalyst bed. Some of the studies cited state that the optimal catalyst bed composition consists of 50% CZA^24,50,64^ while other authors, like us, came to the conclusion that higher CZA-to-acid catalyst ratios are advantageous for the DME productivity.^15,21,46^ To demonstrate the influence of higher CZA-to-γ-Al_2_O_3_ ratios on the reaction rates, these are depicted in Fig. 9 for the reference CZA-to-γ-Al_2_O_3_ weight ratio *μ* = 1, and for *μ* = 2, which exhibited the best performance with regard to the DME yield in the experiments. The increased *μ* is clearly advantageous for all the reactions rates, as assumed in Section 4.1. The effect of the catalyst bed composition is less pronounced than that of the COR and the temperature, and no significant changes on the shapes of the reaction rate profiles is observed. With regard to the concentration profiles, an increased *μ* leads to higher methanol and DME concentrations, whereas the concentration of water is virtually unchanged. Moreover, the relative increase in methanol concentration is higher than the relative increase in DME, indicating a decrease of the selectivity towards DME, consistent with experimental observations described in Section 4.1.

Table S3 in the ESI† provides an overview on studies with different CZA-to-γ-Al_2_O_3_ ratios. A direct comparison with other works regarding this variable is not comprehensively possible, due to the wide range of process parameters evaluated in literature studies,^24^ and also due to more or less widespread catalyst properties, reactor types and configurations, and finally the respective methodology followed in each study. Commonly drawn conclusions in accordance with our work are as follows: (1) DME selectivity increases with decreasing CZA-to-γ-Al_2_O_3_ ratios when CO_2_ is present in the feed.^24,50^ (2) However, decreasing CZA-to-γ-Al_2_O_3_ ratio especially below a value of 1, is detrimental for the DME production.^15,24,64^ (3) Hence, increased DME yield attained with increasing CZA-to-γ-Al_2_O_3_ ratios is attributed to a significant enhancement of the CO_*x*_ conversion, that makes up for the selectivity loss. Higher amounts of the CZA catalyst, evidently lead to higher rates of CO_2_ hydrogenation and water gas shift reaction (Fig. 9a and c), which are both promoted by this catalyst. On the other hand, increased methanol formation and water depletion rates are contributing to methanol dehydration to DME. Hence, explaining the higher rate of the dehydration reaction (Fig. 9b), even though compared to the reference case (*μ* = 1), the fraction of the dehydration catalyst at *μ* = 2 is reduced. It should also be noted that most of the studies mentioned are experimental in scope. This emphasizes the general importance and necessity of models valid for a broader range of catalyst bed compositions (especially also for a wide range of CO_2_/CO_*x*_ feed ratios) to enable model-based evaluation of optimization strategies and/or reactor designs under consideration of these variables.

##### 4.2.2.4 CO_2_ and CO formation during reaction

According to eqn (27), a negative conversion (*X*_*i*_) indicates that the amount of the respective species *i* is higher at the reactor outlet than at the reactor inlet, *i.e.*, that the species was formed during reaction. Within the wide operational windows studied in this work, CO_2_ and CO formation was observed at specific conditions.

**Fig. 10:**
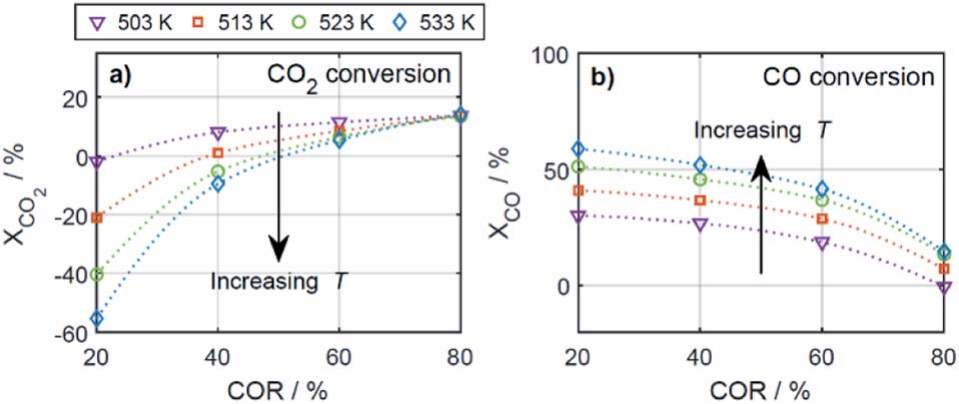
CO_2_ and CO conversion for all evaluated CORs and temperatures. *μ* = 2.

As depicted in Fig. 10a, CO_2_ formation was evidenced at high temperatures and low CORs. The highest CO_2_ formation, *i.e.*, the lowest CO_2_ conversion, was observed at 20% COR and 533 K. At these conditions, the WGSR is faster than the CO_2_ hydrogenation for most of the reactor length. Hence, more CO_2_ is produced than consumed, explaining the negative CO_2_ conversions. Contrary to the results at higher temperatures, CO_2_ formation does not take place at 503 K.

CO formation on the other hand, was evidenced at low temperatures and high CORs (Fig. 10b). The minimal CO conversion took place at 80% COR and 503 K, caused by a relatively late shift of the rWGSR to WGSR. At these conditions, the rWGSR prevailed over the WGSR for approx. half of the reactor length. The CO produced in the first half of the reactor, is not completely consumed in the second half, leading to the slight overall CO production shown in Fig. 10b. In agreement with the mechanistic analysis presented before, CO_2_ and CO conversion show opposite trends, with the CO conversion increasing with temperature, as methanol formation takes place *via* CO hydrogenation.^56^ CO conversion is also increasing with decreasing COR, due to WGSR that is favored at high CO feed concentration, and decreases respectively with increasing COR according to an increased participation of the rWGSR.

## 5. Summary and conclusions

The reaction kinetics of the direct DME synthesis over Cu/ZnO/Al_2_O_3_ (CZA) and γ-Al_2_O_3_ were investigated at high pressure (50 bar) in a temperature range between 503 and 533 K, CZA-to-γ-Al_2_O_3_ weight ratios from 1 to 5, space times from 240 to 400 kg_cat_ s m_gas_^−3^, and carbon oxide ratios (CO_2_/CO_*x*_) from 20 to 80%. The successful fitting to these data resulted in the main contribution of this paper: a mechanistically sound reaction kinetic model with a particularly large range of validity. Due to its wide validity range, the reaction kinetic model provided in this contribution is suitable aiming towards optimal reactor and/or process design, and optimization of novel technologies for the direct DME synthesis.

The influence of key process variables on reaction rates was examined in light of the derived model, and representative results were presented with the goal of determining causality and providing a comprehensive understanding of the observed phenomena. An increased CZA-to-γ-Al_2_O_3_ ratio was found to be favorable in terms of DME yield, although this reduced the amount of dehydrogenation catalyst. This is attributed to the synergistic effects of direct DME synthesis, *i.e.*, an increased methanol production rate also accelerates the dehydration of methanol to DME. With regard to the composition of the feed, a high CO content leads to an increased DME yield, since the water gas shift reaction and thus the water consumption in the system are accelerated. Conversely, a high CO_2_ content leads to a significantly increased water concentration. This is due to a strong effect of reverse water gas shift at the reactor inlet, which increases with CO_2_ content. Moreover, it was shown that increasing temperatures lead to higher DME yield and selectivity regardless of the feed composition. However, at high CO_2_ content in the feed, the attainable enhancement by optimization of the reaction conditions might not lead to sufficiently high DME yields for the process to be economically feasible. Therefore, additional technical improvements are necessary to achieve a significant increase in overall performance. Possible technical improvements include water removal, novel reactor concepts such as membrane reactors or reactive distillation, as well as a customized product separation.

## Conflicts of interest

There are no conflicts to declare.

## Supplementary Material

RA-011-D1RA03452A-s001
