# Supplementary material for: Kinetics of the direct DME synthesis from CO2 rich syngas under variation of the CZA-to-γ-Al2O3 ratio of a mixed catalyst bed
Source: RSC Adv. 2021 Jul 13;11(40):24556–69. doi: 10.1039/d1ra03452a (PMC9036900; doi:10.1039/d1ra03452a)
Supplement: RA-011-D1RA03452A-s001 [file RA-011-D1RA03452A-s001.pdf]

Electronic Supplementary Material (ESI) for RSC Advances.
This journal is © The Royal Society of Chemistry 2021
Electronic Supplementary Material (ESI) for RSC Advances.
This journal is © The Royal Society of Chemistry 2021

ARTICLE

Electronic Supplementary Information

Kinetics of Direct DME Synthesis from CO_2 rich syngas under variation of the CZA-to-γ-Al_2O_3 ratio of a mixed catalyst bed

Nirvana Delgado Otalvaro,[a] Gerardo Sogne,[a] Karla Herrera Delgado,*[a] Stefan Wild,[a] Stephan Pitter[a] and Jörg Sauer[a]

S1. Reduction procedure and conditioning

Before performing the kinetic measurements, the CZA share of the catalytic bed was activated at atmospheric pressure with a volume flow of 300 ml min^{-1} containing 5% of H_2 and 95% of N_2. The system was heated from 373 to 473 K at a heating rate of 20 K h^{-1}. This temperature was hold for one hour, followed by further heating to 513 K at a heating rate of 12 K h^{-1}. Finally, the H_2 concentration in the gas flow was increased to 50%, maintaining the same total flow rate for an additional hour. Posterior to the catalyst reduction, the operating conditions 300 ml min^{-1}, 503 K, 20% COR and 50 bar, were set to allow the catalyst system to run in. This operating point was maintained and the concentration of the product gas was monitored until a steady state of the catalyst system could be assumed (between 12 and 20 h time on stream).

S2. *A priori* Criteria.

Table S1. Calculated criteria for the verification of assumptions.

Phenomena to be neglected	Criteria	Equation	Calculated Value*
Outer mass transfer	Mears[1]	$\eta\ Da_{II} = \frac{r_{j,eff}}{\beta_i\ c_i} < \frac{0.05}{\lvert n \rvert}$	0.0182
Inner mass transfer	Weisz-Prater[2]	$\psi = \frac{(r_{j,eff}\ \ l_c^2)}{D_{i,eff}\ \ c_{i,s}} \frac{n+1}{2} < 0.15$	9.30E-06
Outer heat transfer	Mears[1]	$\frac{\lvert \Delta H_R \rvert\ r_{j,eff}\ R}{\lambda\ T} \frac{E_A}{R\ T} < 0.15$	0.0008
Inner heat transfer	Anderson[3]	$\frac{\lvert \Delta H_R \rvert\ r_{j,eff}\ r_{kat}^2}{\lambda\ T_s} \frac{E_A}{R\ T} < 0.75$	0.0397
Radial Gradients	d/D-ratio[4]	$24 < \frac{d_{Tube}}{d_{Particle}} < 48$	24
Non-Isothermal operation	Rule of Thumb	$\Delta \mathrm{T} = T_{max} - T_{min}$	2
Axial dispersion	Bodenstein Number[5]	$Bo = \frac{u_0\ L}{D_{ax}} > 100$	481
Pressure drop	Δp/L Zhavoronkov Correlation[6]	$\frac{\Delta p_{max}}{p} \sim 0$	5.0 E-08

*for the worst-case scenario

[a] *Karlsruher Institute of Technology (KIT). Hermann-von-Helmholtz-Platz 1, D-76344 Eggenstein-Leopoldshafen, Germany. E-mail: karla.herrera@kit.edu. Tel: +49 721 608 28631*

Please do not adjust margins

S3. Model Discrimination.

For the initial model discrimination, the available experimental data were first simulated using eight different kinetic models from the open literature[7–14]. The sum of the squared errors between the measured and predicted composition of the product gas was calculated for each model and depicted in Fig. S1. In this figure, the models are named after the first author.

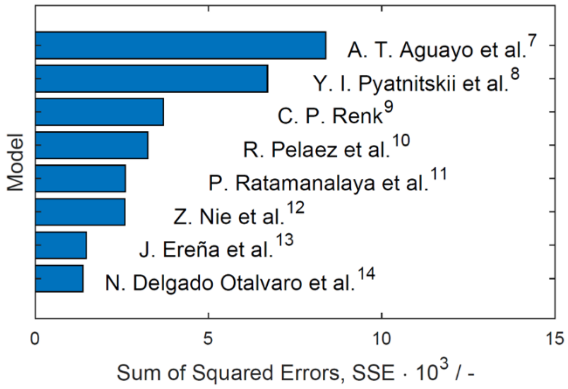

Figure S1. Total sum of squared errors for the implemented models

After this initial screening, the five models with the lower residual squared sum were parametrized to fit the experimental data. The model by Delgado Otalvaro et al.[14] agreed best with the available experimental data. Hence, the model structure and respective mechanistic assumptions were chosen for fine-tuning. To enable a direct comparison of the tested models and parameters, these have been compiled in Table S2. Additionally, the mean relative error between the predictions with the different models, and the experiments for each species i (RE_i) is also given. RE_i is calculated by:

$$RE_i = 100\% \quad \frac{1}{No.Exps} \sum_{n=1}^{No.Exps} \frac{\left|y_{i,out,measured,n} - y_{i,out,predicted,n}\right|}{y_{i,out,measured,n}}.$$

The indices of the reaction rates, and rate constants in Table S2 correspond to the following reactions:

1. $CO_2 + 3\,H_2 \rightleftharpoons CH_3OH + H_2O$
2. $2\,CH_3OH \rightleftharpoons CH_3OCH_3 + H_2O$
3. $CO + H_2O \rightleftharpoons CO_2 + H_2$
4. $CO + 2\,H_2 \rightleftharpoons CH_3OH$
5. $n CO + (2n+1) H_2 \rightleftharpoons C_nH_{2n+2} + n H_2O$

Table S2. Compilation of tested reaction kinetic models with the respective specific parameters, and resulting relative error for each species.

Model	Rate expressions	Model specific parameter	RE/%
N. Delgado Otalvaro[14]	$r_1 = k_1 \frac{\left(f_{CO_2} f_{H_2}^3 - \frac{f_{H_2O} f_{CH_3OH}}{K_{f,1}}\right)[(1-\varepsilon_{bed})\rho_{CZA}\,\xi_{CZA}]}{\left(1 + K_{CO_2} f_{CO_2} + K_{CO} f_{CO} + \sqrt{K_{H_2} f_{H_2}}\right)^3}$<br>$r_2 = k_2 \left(f_{CH_3OH}^2 - \frac{f_{DME} f_{H_2O}}{K_{f,2}}\right)[(1-\varepsilon_{bed})\rho_{ALOX}\,\xi_{ALOX}]$<br>$r_3 = k_3 \frac{\left(f_{H_2O} - \frac{f_{CO_2} f_{H_2}}{K_{f,3} f_{CO}}\right)[(1-\varepsilon_{bed})\rho_{CZA}\,\xi_{CZA}]}{1 + K_{CO_2} f_{CO_2} + K_{CO} f_{CO} + \sqrt{K_{H_2} f_{H_2}}}$	$k_1 = \exp(-6.94)\exp\left[-\frac{21.81}{R\,T_R}\left(\frac{T_R}{T}-1\right)\right]$<br>$k_2 = \exp(-2.07)\exp\left[-\frac{42.77}{R\,T_R}\left(\frac{T_R}{T}-1\right)\right]$<br>$k_3 = \exp(-2.75)\exp\left[-\frac{10.82}{R\,T_R}\left(\frac{T_R}{T}-1\right)\right]$<br>$K_{CO} = \exp(-15.32)\exp\left[\frac{14.03}{R\,T_R}\left(\frac{T_R}{T}-1\right)\right]$<br>$K_{CO_2} = \exp(-0.57)\exp\left[-\frac{0}{R\,T_R}\left(\frac{T_R}{T}-1\right)\right]$<br>$K_{H_2} = \exp(-19.51)\exp\left[\frac{14.68}{R\,T_R}\left(\frac{T_R}{T}-1\right)\right]$<br>$[r_j] = \mathrm{mol\,m^{-3}s^{-1}}$	CO<br>9.7<br>CO2<br>7.1<br>H2<br>1.5<br>DME<br>54.5
J. Ereña[13]	$r_2 = k_2 \left(f_{CH_3OH}^2 - \frac{f_{DME} f_{H_2O}}{K_2}\right)$	$k_2 = 3.41 \cdot 10^{-3} \exp\left(-\frac{63.5}{R}\left(\frac{1}{T}-\frac{1}{548}\right)\right)$	CO<br>11.3

 This journal is © The Royal Society of Chemistry 20xx

Please do not adjust margins

	$r_3 = k_3\left(f_{CO}f_{H_2O} - \frac{f_{CO_2} f_{H_2}}{K_3}\right)$<br>$r_4 = k_4 \frac{\left(f_{H_2}^2 f_{CO} - \frac{f_{CH_3OH}}{K_4}\right)\frac{r_{CH_3OH}}{(r_{CH_3OH})_0}}{(1 + K_{H_2O} f_{H_2O} + K_{CO_2} f_{CO_2})}$<br>$r_5 = k_5 \frac{\left(f_{CO}f_{H_2}^3 - \frac{f_{HC} f_{H_2O}}{K_5}\right)}{(1 + K_{H_2O} f_{H_2O} + K_{CO_2} f_{CO_2})}$	k_3: not given<br>$k_4 = 2.4 \cdot 10^{-5} \exp\left(-\frac{90.0}{R}\left(\frac{1}{T}-\frac{1}{548}\right)\right)$<br>$k_5 = 1.23 \cdot 10^{-7} \exp\left(-\frac{9.9}{R}\left(\frac{1}{T}-\frac{1}{548}\right)\right)$<br>$K_{H_2O} = 2.67 \cdot 10^{-3} \exp\left(\frac{384.8}{R}\left(\frac{1}{T}-\frac{1}{548}\right)\right)$<br>$K_{CO_2} = 1.13 \cdot 10^{-3} \exp\left(\frac{43.4}{R}\left(\frac{1}{T}-\frac{1}{548}\right)\right)$<br><br>$[k_2] = mol_{DME}\ (mol_c)^{-1} g_{cat}^{-1}\ h^{-1} bar^{-2}$<br>$[k_4] = mol_{CH_3OH} (mol_c)^{-1} g_{cat}^{-1}\ h^{-1} bar^{-3}$<br>$[k_5] = mol_{HC} (mol_c)^{-1} g_{cat}^{-1}\ h^{-1} bar^{-4}$<br>$[E_a] = kJ\ mol^{-1}$<br>$[\Delta H_{ads}] = kJ\ mol^{-1}$<br>$[K_i] = bar^{-1}$	CO2<br>7.6<br><br>H2<br>1.6<br><br>DME<br>62.8
Z. Nie[12]	$r_1 = k_1 \frac{f_{CO_2} f_{H_2}^3 \left(1 - \frac{f_{CH_3OH} f_{H_2O}}{K_{f_1} f_{CO_2} f_{H_2}^3}\right)}{(1 + K_{CO} f_{CO} + K_{CO_2} f_{CO_2} + K_{H_2} f_{H_2})^4}$<br>$r_2 = k_2 \frac{f_{CH_3OH} \left(1 - \frac{f_{DME} f_{H_2O}}{K_{f_2} f_{CH_3OH}^2}\right)}{\left(1 + \sqrt{K_{CH_3OH} f_{CH_3OH}}\right)^2}$<br>$r_4 = k_4 \frac{f_{CO} f_{H_2}^2 \left(1 - \frac{f_{CH_3OH}}{K_{f_4} f_{CO} f_{H_2}^2}\right)}{(1 + K_{CO} f_{CO} + K_{CO_2} f_{CO_2} + K_{H_2} f_{H_2})^3}$	$k_1 = 5.059 \cdot 10^3 \exp\left(-\frac{67515}{R\,T}\right)$<br>$k_2 = 1.602 \cdot 10^3 \exp\left(-\frac{43473}{R\,T}\right)$<br>$k_4 = 7.380 \cdot 10^3 \exp\left(-\frac{54307}{R\,T}\right)$<br>$K_{CO} = 3.934 \cdot 10^{-6} \exp\left(\frac{37373}{R\,T}\right)$<br>$K_{CO_2} = 1.858 \cdot 10^{-6} \exp\left(\frac{53795}{R\,T}\right)$<br>$K_{H_2} = 0.6716 \exp\left(-\frac{6476}{R\,T}\right)$<br>$K_{CH_3OH} = 3.480 \cdot 10^{-6} \exp\left(\frac{54689}{R\,T}\right)$<br><br>$[r_j] = ml\ g^{-1} h^{-1}$	CO<br>36.5<br><br>CO2<br>10.6<br><br>H2<br>6.7<br><br>DME<br>100
P. Ratamanalaya[11]	$r_2 = k_2 \frac{\left(p_{CH_3OH}^2 - \frac{p_{DME}\, p_{H_2O}}{K_{eq_2}}\right)}{\left(1 + \sqrt{K_{CH_3OH}\, p_{CH_3OH}} + K_{H_2O}\, p_{H_2O}\right)^2}$<br>$r_3 = k_3 \frac{\left(p_{CO} p_{H_2O} - \frac{p_{CO_2}\, p_{H_2}}{K_{eq_3}}\right)}{\left(1 + K_{CO}\, p_{CO} + K_{H_2O}\, p_{H_2O} + K_{CO_2}\, p_{CO_2} + \sqrt{K_{H_2}\, p_{H_2}}\right)^2}$<br>$r_4 = k_4 \frac{\left(p_{CO}\, p_{H_2}^2 - \frac{p_{CH_3OH}}{K_{eq_4}}\right)}{\left(1 + K_{CO}\, p_{CO} + \sqrt{K_{H_2}\, p_{H_2}} + K_{CH_3OH}\, p_{CH_3OH}\right)^3}$	$k_2 = 1.69 \cdot 10^8 \exp\left(-\frac{69787}{R\,T}\right)$<br>$k_3 = 1202.8 \exp\left(-\frac{20437}{R\,T}\right)$<br>$k_4 = 40.498 \exp\left(-\frac{18203}{R\,T}\right)$<br>$K_{CH_3OH} = 0.9535 \exp\left(\frac{16243}{R\,T}\right)$<br>$K_{H_2O} = 6.992 \exp\left(\frac{26452}{R\,T}\right)$<br>$K_{CO} = 4.49 \cdot 10^{-7} \exp\left(\frac{60528}{R\,T}\right)$<br>$K_{CO_2} = 1.092 \cdot 10^{-7} \exp\left(\frac{66924}{R\,T}\right)$<br>$K_{H_2} = 0.2487 \exp\left(\frac{30961}{R\,T}\right)$<br><br>$[K_i] = bar^{-1}$	CO<br>36.1<br><br>CO2<br>10.7<br><br>H2<br>6.7<br><br>DME<br>100
R. Pelaez[10]	$r_1 = \frac{k_1}{(1 + K_{H_2O} f_{H_2O})^3}\left(f_{CO_2} f_{H_2} - \frac{f_{CH_3OH} f_{H_2O}}{K_{eq_1} f_{H_2}^2}\right)$<br>$r_2 = k_2\left(f_{CH_3OH}^2 - \frac{f_{DME} f_{H_2O}}{K_{eq_2}}\right)$<br>$r_3 = k_3\left(f_{CO} f_{H_2O} - \frac{f_{CO_2} f_{H_2}}{K_{eq_3}}\right)$<br>$r_4 = \frac{k_4}{(1 + K_{H_2O} f_{H_2O})^3}\left(f_{CO} f_{H_2} - \frac{f_{CH_3OH}}{K_{eq_4} f_{H_2}}\right)$	Equation for temperature dependency of the rate or adsorption constants is not given. Parameter estimates:<br><br>$k_{1,at\ 523\ K} = 2.55 \cdot 10^{-3}\ mol\ kg_{cat}^{-1}\ s^{-1} bar^{-2}$<br>$E_{a,1} = 3.8\ kJ/mol$<br>$k_{2,at\ 523\ K} = 8.13\ mol\ kg_{cat}^{-1}\ s^{-1} bar^{-2}$<br>$k_3$: not given ($r_3$ at equilibrium)<br>$k_{4,at\ 523\ K} = 6.43 \cdot 10^{-7} mol\ kg_{cat}^{-1}\ s^{-1} bar^{-2}$<br>$E_{a,4} = 171.8\ kJ/mol$<br>$K_{H_2O} = 19\ bar^{-1}$	CO<br>25.8<br><br>CO2<br>13.2<br><br>H2<br>6.4<br><br>DME<br>99.5

C. P. Renk[9]	$r_2 = k_2\, c_{CH_3OH}^{1,1} - k_{2'}\, c_{DME}^{1,2}\, c_{H_2O}$<br>$r_3 = k_3\, c_{CO}^{0,2} c_{H_2O}^{1,3} - k_{3'}\, c_{H_2}^{0,4}\, c_{CO_2}^{0,2}$<br>$r_4 = k_4\, c_{H_2}\, c_{CO}^{0,5} - k_{4'}\, c_{CH_3OH}^{2,4}$	$k_2 = 5.1 \cdot 10^1 \exp\left(-\frac{0}{R\,T}\right)$<br>$k_{2'} = 8.6 \cdot 10^6 \exp\left(-\frac{31}{R\,T}\right)$<br>$k_3 = 1.5 \cdot 10^4 \exp\left(-\frac{0.2}{R\,T}\right)$<br>$k_{3'} = 2.6 \cdot 10^9 \exp\left(-\frac{107}{R\,T}\right)$<br>$k_4 = 1.4 \cdot 10^{13} \exp\left(-\frac{138}{R\,T}\right)$<br>$k_{4'} = 1.1 \cdot 10^{11} \exp\left(-\frac{44}{R\,T}\right)$<br><br>$[E_a] = \mathrm{kJ\ mol^{-1}}$<br>$[k_j] = [k_{j'}] = \mathrm{ml\ g^{-1}\ s^{-1}\ ml^{n-1}\ mol^{1-n}}$	CO<br>26.8<br><br>CO2<br>12.4<br><br>H2<br>7.6<br><br>DME<br>100
Y. I. Pyatnitskii[8]	$r_1 = k_1 \frac{p_{CO2}\, p_{H_2}\left(1 - \frac{p_{H_2O}\, p_{CH_3OH}}{K_1\, p_{H_2}^3\, p_{CO_2}}\right)}{\left(1 + a_3 \frac{p_{H_2O}}{p_{H_2}} + a_1 \sqrt{p_{H_2}} + a_2\, p_{H_2O}\right)^3}$<br>$r_2 = k_2 \frac{p_{CH_3OH}\left(1 - \frac{p_{DME}\, p_{H_2O}}{K_2\, p_{CH_3OH}^2}\right)}{1 + K_{CH_3OH}\, p_{CH_3OH} + \frac{p_{H_2O}}{K_{H_2O}}}$<br>$r_3 = -k_3 \frac{p_{CO2}\left(1 - K_3 \frac{p_{H_2O}\, p_{CO}}{p_{H_2}\, p_{CO_2}}\right)}{1 + a_3 \frac{p_{H_2O}}{p_{H_2}} + a_1 \sqrt{p_{H_2}} + a_2\, p_{H_2O}}$	$k_1 = 0.00107 \exp\left(\frac{4414}{T}\right)$<br>$k_2 = 2.82 \cdot 10^6 \exp\left(-\frac{6938}{T}\right)$<br>$k_3 = 1.22 \cdot 10^7 \exp\left(-\frac{11398}{T}\right)$<br>$a_1 = 0.499 \exp\left(\frac{2068}{T}\right)$<br>$a_2 = 6.62 \cdot 10^{-11} \exp\left(\frac{14928}{T}\right)$<br>$a_3 = 3453.38$<br>$K_{CH_3OH} = 2.20 \cdot 10^{-5} \exp\left(\frac{7738}{T}\right)$<br>$K_{H_2O} = 0.051 \exp\left(\frac{626}{T}\right)$<br><br>$[a_1] = \mathrm{bar^{-0.5}}$, $[a_2] = \mathrm{bar^{-1}}$<br>$[a_3] = -$<br>$[k_1] = \mathrm{mol\ g^{-1}\ s^{-1}\ bar^{-2}}$<br>$[k_2] = \mathrm{mol_{DME}\ g^{-1}\ h^{-1}\ bar^{-1}}$<br>$[k_3] = \mathrm{mol\ g^{-1}\ s^{-1}\ bar^{-1}}$<br>$[K_{CH_3OH}] = \mathrm{bar^{-1}}$, $[K_{H_2O}] = \mathrm{bar}$	CO<br>26.6<br><br>CO2<br>18.4<br><br>H2<br>7.3<br><br>DME<br>98.5
A. T. Aguayo[7] (Model 1)	$r_2 = k_2\left(f_{CH_3OH}^2 - \frac{f_{DME}\, f_{H_2O}}{K_2}\right)$<br>$r_3 = k_3\left(f_{CO} f_{H_2O} - \frac{f_{CO_2}\, f_{H_2}}{K_3}\right)$<br>$r_4 = k_4\left(f_{CO}\, f_{H_2}^2 - \frac{f_{CH_3OH}}{K_4}\right)$<br>$r_5 = k_5\left(f_{CO} f_{H_2}^3 - \frac{f_{HC}\, f_{H_2O}}{K_5}\right)$	$k_2 = 1.44 \exp\left(-\frac{80.64}{R}\left(\frac{1}{T} - \frac{1}{548}\right)\right)$<br>$k_3$ not given (r_3 in equilibrium)<br>$k_4 = 1.91 \cdot 10^{-6} \exp\left(-\frac{11.3}{R}\left(\frac{1}{T} - \frac{1}{548}\right)\right)$<br>$k_5 = 2.04 \cdot 10^{-7} \exp\left(\frac{-15.92}{R}\left(\frac{1}{T} - \frac{1}{548}\right)\right)$<br><br>$[E_a] = \mathrm{kcal\ mol^{-1}}$<br>$[k_2] = \mathrm{mol_{DME}(mol_{H_2})^{-1} g_{cat}^{-1}\ h^{-1} bar^{-2}}$<br>$[k_4] = \mathrm{mol_{CH_3OH}(mol_{H_2})^{-1} g_{cat}^{-1}\ h^{-1} bar^{-3}}$<br>$[k_5] = \mathrm{mol_{HC}(mol_{H_2})^{-1} g_{cat}^{-1}\ h^{-1} bar^{-4}}$	CO<br>35.7<br><br>CO2<br>9.9<br><br>H2<br>7.3<br><br>DME<br>127.2

This journal is © The Royal Society of Chemistry 20xx

S4. Selectivity

The selectivity towards DME is displayed here in Figs. S2 and S3 complementary to Figs. 1, 2 and 4 of the manuscript.

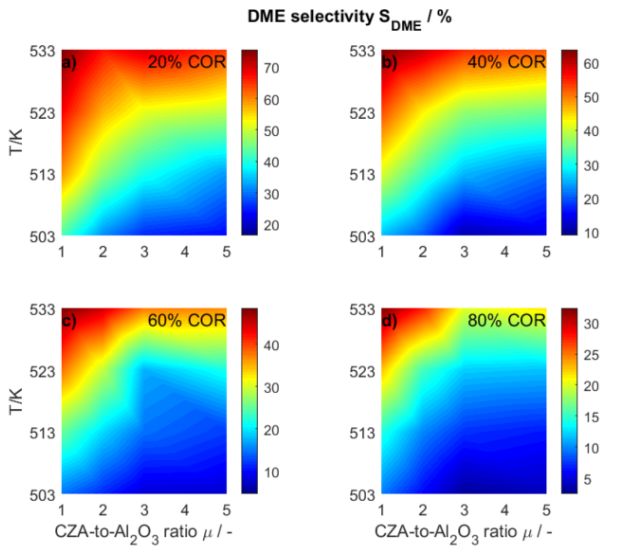

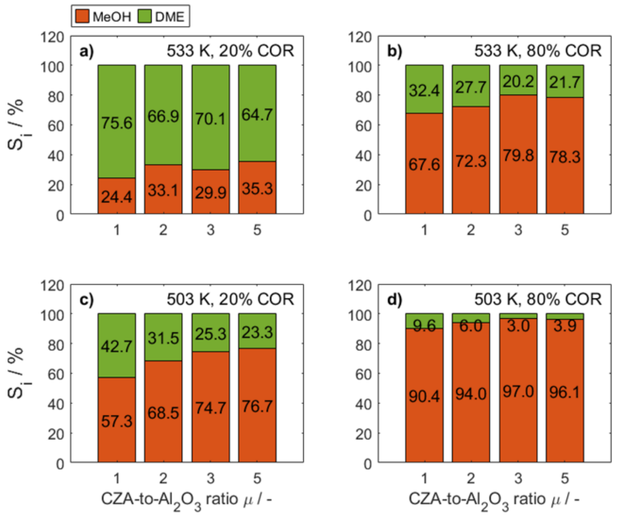

Figure S2. DME selectivity determined experimentally and plotted as a function of the temperature (T) and the CZA-to-γ-Al_2O_3 ratio (μ) for CORs of **a)** 20% **b)** 40% **c)** 60% **d)** 80%. Experimental conditions summarized in Table 1.

Figure S3. Selectivity of methanol and DME at specific conditions: **a)** 533 K, 20% COR, **b)** 533 K, 80% COR, **c)** 492 K, 20% COR **d)** 492 K, 80% COR

S5. Mole percentage profiles including CO and CO_2

The concentration profiles of CO and CO_2 are shown here for the sake of completeness.

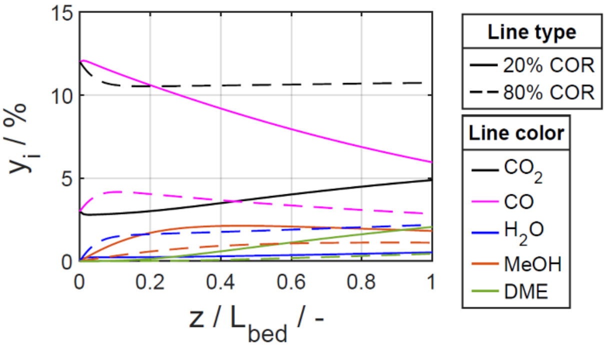

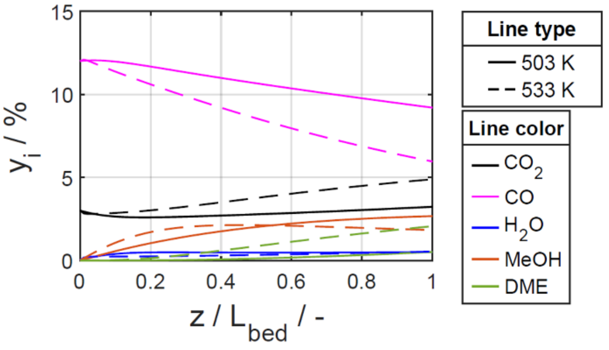

Figure S4. Mole percentage profiles of CO, CO_2, water, methanol and DME at T=533 K, μ=2.
(—) Solid lines: 20% COR, (---) Dashed lines: 80% COR

Figure S5. Mole percentage profiles of CO, CO_2, water, methanol and DME at μ=2 and COR=20%**.**
(—) Solid lines: T=503 K, (---) Dashed lines: T=533 K

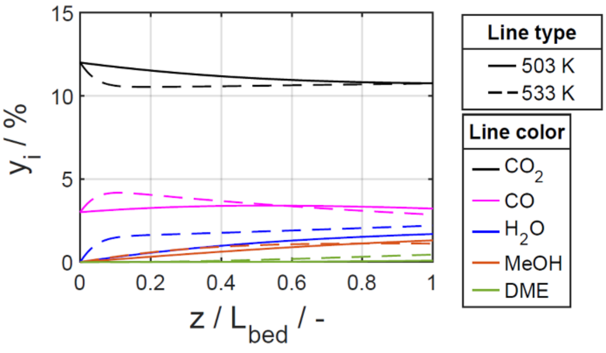

Figure S6. Mole percentage profiles of CO, CO_2, water, methanol and DME at μ=2 and COR=80%.
(—) Solid lines: T=503 K, (---) Dashed lines: T=533 K

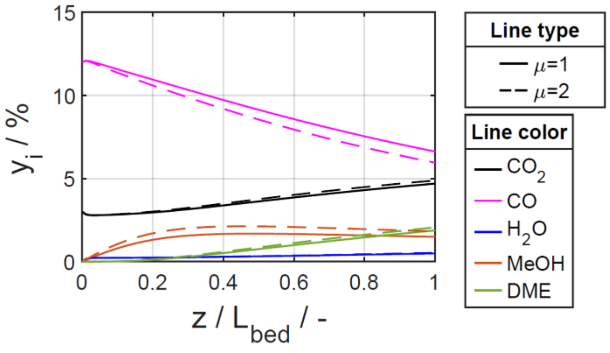

Figure S7. Mole percentage profiles of CO, CO_2, water, methanol and DME at T=533 K, COR=20%.
(—) Solid lines: μ=1, (---) Dashed lines: μ=2

S6. Overview of selected studies conducted at different CZA-to-γ-Al_2O_3 ratios

Table S3. Overview of selected studies conducted at different CZA-to-γ-Al_2O_3 ratios

Study - named after first author	Catalyst system and properties	Feed, conditions, reactor,* catalyst particle size	Optimal catalyst bed composition**
R. Pelaez et al.[10]	- CZA: CHEMPACK<br>S_{BET}: 76.6 m^2g^{-1}<br>Pore volume: 0.257 cm^3g^{-1}<br><br>- γ-Al_2O_3: BASF<br>S_{BET}: 239.9 m^2g^{-1}<br>Pore volume: 0.545 cm^3g^{-1}	$CO/CO_2/H_2$<br>250-270 °C, 30 bar,<br>0.067-0.244 kgh/Nm^3<br>100-250 μm<br>CZA/γ-Al_2O_3: 70/30, 85/15, 92.5/7.5%	CZA/γ-Al_2O_3: 92.5/7.5% regarding CO conversion and DME yield
N. Delgado Otalvaro et al.[14]	Commercial CZA/γ-Al_2O_3	$CO/CO_2/H_2$/Inert<br>220-260 °C, 50 bar,<br>200-700 Nml/min,<br>250-500 μm<br>(model-based optimization)	65.5% CZA,<br>34.5 % γ-Al_2O_3 (v.) without dilution. Regarding conversion of CO_x and DME yield
K. L. Ng et al.[15]	- CZA<br>- γ-Al_2O_3: Norton Chemicals Co.	$CO/CO_2/H_2$/He,<br>250 °C, 50 bar,<br>27500 h^{-1},<br>Gradientless, internal-recycle-type reactor, stacked catalysts,<br>250-500 μm,<br>CZA/γ-Al_2O_3: 1/0, 1/0.5, 1/1, 1/2	CZA/γ-Al_2O_3: 1/2 regarding DME yield***
C. Peinado et al.[16]	-CZA: Katalco 51-8<br><br>- γ-Al_2O_3: Alfa Aesar bimodal<br>S_{BET}: 220-280 m^2g^{-1}	$CO/CO_2/H_2$,<br>270-290 °C, 25-50 bar,<br>5000-7500 h^{-1},<br>250-300 μm,<br>CZA/γ-Al_2O_3: 90/10, 50/50, 10/90	CZA/γ-Al_2O_3: 50/50 regarding DME productivity
J. W. Bae et al.[17]	- CZA: prod. in-house<br>$CuO/ZnO/Al_2O_3$=50/40/10<br><br>- γ-Al_2O_3: prod. in-house<br>S_{BET}: 437.8 m^2g^{-1}	$CO/CO_2/H_2$: 41/21/38 v. %,<br>250 °C, 40 bar,<br>11000 h^{-1}<br>Pellet form hybrid catalyst<br>CZA/γ-Al_2O_3: 1, 3, 5	CZA/γ-Al_2O_3: 1<br>Regarding the DME selectivity<br><br>CZA/γ-Al_2O_3: 5<br>Regarding the CO conversion
J. Abu-Dahrieh et al.[18]	-CZA: prod. in-house<br>$CuO/ZnO/Al_2O_3$=60/30/10<br>S_{BET}: 56.9 m^2g^{-1}<br>pore size: 1.05 nm	$CO/CO_2/H_2/Ar$: 31/4/62/3 v. %,<br>200-260 °C, 20 bar,<br>2400 ml $g^{-1}h^{-1}$,<br>250-425 μm,	CZA/γ-Al_2O_3: 1<br>and<br>CZA/HZSM-5: 3<br>regarding the DME yield

This journal is © The Royal Society of Chemistry 20xx

Please do not adjust margins

	- NH4ZSM-5,<br>HZSM-5 and<br>γ-Al_2O_3<br><br>γ-Al_2O_3: prod. in-house,<br>S_{BET}: 117 m^2g^{-1},<br>Pore size = 1.035 nm	admixed catalyst,<br>CZA/γ-Al_2O_3: 1, 2, 3	
A. Ateka et al.[19]	Comparison of different commercial and in-hose made catalyst systems<br><br>- CuO–ZnO–ZrO2,<br>CuO–ZnO–MnO, and<br>CuO/ZnO/Al_2O_3<br><br>CZA: S_{BET}: 24 m^2g^{-1},<br>Pore volume: 0.081 cm^3g^{-1}<br>Cu dispersion: 5.2%<br><br>- SAPO-18 and γ-Al_2O_3<br><br>SAPO-18: S_{BET}: 480 m^2g^{-1},<br>Pore volume: 0.39 cm^3g^{-1}<br>Total acidity: 0.42 $mmol_{NH3}/g_{cat}$	CO/CO_2/H_2,<br>275 °C, 30 bar,<br>3.7 g h $(mol_C)^{-1}$,<br>125-500 µm,<br>Bifunctional catalysts<br>CZA/SAPO-18: 1, 2, 5, 10	CZA/SAPO-18: 2 regarding yield and selectivity of DME for CO_2 free feeds (H_2/CO=3)

* The reactor type is a fixed bed tubular reactor, with mechanically mixed catalyst bed unless otherwise stated
** percentages and ratios in weight, unless otherwise stated
*** In this study the mass of CZA was held constant while the mass of γ-Al_2O_3 was increased to achieve a higher γ-Al_2O_3-to-CZA ratio. Hence, the γ-Al_2O_3-to-CZA ratio leading to the highest performance was the case at which the total catalyst mass was also the highest.

Notes and references

1 D. Mears, *J. Catal.*, 1971, **20**, 127–131.
2 P. B. Weisz and C. D. Prater, in *Advances in Catalysis*, eds. W. G. Frankenburg, V. I. Komarewsky and E. K. Rideal, Academic Press, 1954, pp. 143–196.
3 J. B. Anderson, *Chem. Eng. Sci.*, 1963, **18**, 147–148.
4 J. Pérez-Ramírez, *Catal. Today*, 2000, **60**, 93–109.
5 M. Baerns, A. Behr, A. Brehm, J. Gmehling, H. Hofmann and U. Onken, *Technische chemie*, John Wiley & Sons, 2013.
6 T. Zeiser, Friedrich-Alexander-Universität Erlangen-Nürnberg, 2008.
7 A. T. Aguayo, J. Ereña, D. Mier, J. M. Arandes, M. Olazar and J. Bilbao, *Ind. Eng. Chem. Res.*, 2007, **46**, 5522–5530.
8 Y. I. Pyatnitskii, P. E. Strizhak and N. K. Lunev, *Theor. Exp. Chem.*, 2009, **45**, 325–330.
9 C. P. Renk, Forschungszentrum Karlsruhe GmbH, 2009.
10 R. Peláez, P. Marín and S. Ordóñez, *Fuel Process. Technol.*, 2017, **168**, 40–49.
11 P. Ratamanalaya, S. Limtrakul, T. Vatanatham and P. Ramachandran, in *TIChE International Conference 2011*, 2011, p. ee014.1-ee014.5.
12 Z. Nie, H. Liu, D. Liu, W. Ying and D. Fang, *J. Nat. Gas Chem.*, 2005, **14**, 22–28.
13 J. Ereña, I. Sierra, A. T. Aguayo, A. Ateka, M. Olazar and J. Bilbao, *Chem. Eng. J.*, 2011, **174**, 660–667.
14 N. Delgado Otalvaro, M. Kaiser, K. Herrera Delgado, S. Wild, J. Sauer and H. Freund, *React. Chem. Eng.*, 2020, **5**, 949–960.
15 K. L. Ng, D. Chadwick and B. A. Toseland, *Chem. Eng. Sci.*, 1999, **54**, 3587–3592.
16 C. Peinado, D. Liuzzi, M. Retuerto, J. Boon, M. A. Peña and S. Rojas, *Chem. Eng. J. Adv.*, 2020, **4**, 100039.
17 J. W. Bae, H. S. Potdar, S. H. Kang and K. W. Jun, *Energy and Fuels*, 2008, **22**, 223–230.
18 J. Abu-Dahrieh, D. Rooney, A. Goguet and Y. Saih, *Chem. Eng. J.*, 2012, **203**, 201–211.
19 A. Ateka, I. Sierra, J. Ereña, J. Bilbao and A. T. Aguayo, *Fuel Process. Technol.*, 2016, **152**, 34–45.

This journal is © The Royal Society of Chemistry 20xx

Please do not adjust margins
